# The use of individual tracking programs in public health: a bioethics dilemma

**DOI:** 10.1590/0034-7167-2023-0041

**Published:** 2024-08-19

**Authors:** Ricardo Jardim Neiva, João Felício Rodrigues, Ana Paula Oliveira Nascimento Alves, Patrícia de Sousa Fernandes Queiroz, Leila das Graças Siqueira, Clara Cynthia Melo e Lima, Francisca Souza Santos Dias, Renê Ferreira da Silva-Júnior

**Affiliations:** I Instituto Federal do Norte de Minas Gerais. Araçuaí, Minas Gerais. Brazil; II Universidade Estadual de Montes Claros. Montes Claros, Minas Gerais. Brazil; III Instituto Federal do Norte de Minas Gerais. Januária, Minas Gerais. Brazil; IV Instituto Federal do Sul de Minas Gerais. Machado, Minas Gerais. Brazil

**Keywords:** Bioethics, Contact Tracing, Public Health, Ethical Theory, Universities, Bioética, Tamizaje Masivo, Salud Pública, Revisión Ética, Universidades

## Abstract

**Objective:**

to understand the bioethical perspectives on mobile tracking device use.

**Methods:**

theoretical study based on action research, carried out with eight graduate students from a public university. A focus group was used, with a thematic content analysis methodology with a codebook structure, approved by the Research Ethics Committee.

**Results:**

from the analysis, there was a concern about using devices after the pandemic ended. Using or not the device, rights inherent to humans, legislation and effectiveness of methods deepen interpretations, moving participants from a personalistic conception of the topic to a vision focused on professional implications about the methods.

**Final considerations:**

the debate on the impact of using technological devices on health, especially those that imply restriction of rights that refer to individuals’ private life, involves a discussion of a professional nature, in addition to requirement for clear rules on the topic.

## RESUMEN

**Objetivo:**

comprender las perspectivas bioéticas sobre el uso de dispositivos móviles de seguimiento.

**Métodos:**

estudio teórico basado en la investigación acción, realizado con ocho estudiantes de posgrado de una universidad pública. Se utilizó un grupo focal, con metodología de análisis de contenido temático con estructura de libro de códigos, aprobada por el Comité de Ética en Investigación.

**Resultados:**

del análisis surgió la preocupación por el uso de dispositivos una vez terminada la pandemia. El uso o no del dispositivo, los derechos inherentes a la persona humana, la legislación y la eficacia de los métodos profundizan las interpretaciones, trasladando a los participantes de una concepción personalista del tema a una visión centrada en las implicaciones profesionales de los métodos.

**Consideraciones finales:**

el debate sobre el impacto del uso de dispositivos tecnológicos en la salud, especialmente aquellos que implican restricción de derechos que se refieren a la vida privada de las personas, implica una discusión profesional, además de la exigencia de reglas claras sobre el tema.

## INTRODUCTION

Bioethics is an area of study and knowledge that seeks, above other concepts, to defend life, the environment and health. To this end, it uses its academic, plural and non-corporate structure, focusing on issues sensitive to human life. The term has transformed over time into a means of communicating various areas of knowledge, in moral, ethical, religious, technical-scientific and other issues, with the intention of outlining strategies for protecting individuals^(1-2)^.

In the historical path of bioethics in principialist logic, society proposes discussions in the area on topics such as autonomy, beneficence, non-maleficence and justice^(3)^. Therefore, it should be emphasized that the interventionist version encompasses areas such as geopolitics, global emergence and re-emergence, equity and equality, among others. Bioethics also deals with human sciences, especially sociology, on topics such as “epistemological integrity of health sciences, ethical-political analyzes around access and security of new and old health technologies”^(4)^.

In healthcare evolution, it is necessary to constantly reflect on using technological devices to meet structural demands so that there is no negative influence and/or harm to the community. Data production and use in digital technologies continues to advance from the second decade of the 21^st^ century, increased following the COVID-19 pandemic^(5-7)^. Computational modeling and algorithm use provide a new way of producing knowledge, which is increasingly used. Data has gained relevance for personal, commercial, economic, political and social relationships. It is necessary that good quality information is available to professional teams for basic analysis of epidemiological data and to guide information and clinical measure production for diagnosis, management, in addition to new case rehabilitation and prevention^(8)^.

Due to the lack of prospects for a large-scale vaccine blockade in the initial months of the pandemic, software companies began work on improving tools for the specific case of identifying contacts of the disease. This is a new modality of epidemiological control, although aspects of its applicability have not yet been properly analyzed^(9-11)^.

In Brazil, the application used was the API Exposure Notification, made available free of charge by the Ministry of Health in partnership with technology companies Apple^®^ and Google^®^. It came into force in August 2021 on the Coronavirus-SUS platform. In 2021, a booklet was published that pays attention to the regulation of use of individual data in tracking devices^(12)^. The document seeks to make data protection regulations and individual monitoring compatible with public interests and legal security maintenance. The General Individual Data Protection Law, sanctioned in 2018, must therefore be applied to the logic of individual data in the pandemic. The technology used in Brazil would be data decentralization, with the files reserved for the device of the person who installed the application^(13)^. In the same step, Ordinance 2358 of September 2, 2020 creates financial and operational incentives to boost contact tracing and monitoring^(14)^. This allows municipalities to autonomously create information systems for tracking records, as long as they are interoperable with the federal system *e-SUS Notifica*. After the critical period of the pandemic, studies show that the system compulsorily synchronized data on suspected cases of COVID-19, with a high number of incomplete and late information for effective actions^(5)^. Such an act puts at risk concepts of data security, the right to anonymity and privacy. Returning to the principialist basis of bioethics in Brazil, there is a flagrant conflict between acts regarding autonomy, non-maleficence and justice.

Health surveillance and monitoring are practices carried out by the democratic state and guaranteed by laws that separate individual and collective interests. COVID-19, however, promoted a collision between the right to privacy and fundamental rights to health and life, requiring the establishment of balancing guidelines^(5, 15)^.

Tracking technology use crosses bioethics in dimensions that range from personal to collective. It is one of the new paradigms in the area, also approached as one of the possible uses of Artificial Intelligence (AI), including genetic engineering and telemedicine^(15)^. Considering the above, we sought to understand the personal understanding of graduate students from a public institution working in the health sector regarding bioethical issues involving tracking device use. Bioethical aspects involve personal monitoring, with restrictions on individual freedoms for the benefit of a community in a life-threatening epidemic situation.

The topic in question does not present a global consensus, but it directs guiding elements of bioethical thinking. Among them, we can highlight the conflict between privacy and individual freedom, obligations involved and personal and institutional responsibilities over the process^(16)^. Therefore, bioethics encompasses discussions about the scope and implications of monitoring people through technologies, with the argument of preserving collective health.

## OBJECTIVE

To understand personal understanding of the bioethical perspectives involved in the personal and professional use of mobile tracking devices.

## METHODS

### Ethical aspects

The research considered the guidelines of Resolution 510 of April 7, 2016 and Resolution 466 of December 12, 2012 of the Brazilian National Health Council. The Informed Consent Form (ICF) was signed by all participants and researcher in charge. The project was approved under the opinion of the *Universidade Estadual de Montes Claros* Research Ethics Committee, attached to this submission. The transcriptions were prepared using P1, P2, P3... (“P” for participant and numbering according to the completion of the questionnaires, in sequence), in order to guarantee participant anonymity.

### Study design

This is a qualitative study, based on the COnsolidated criteria for REporting Qualitative research (COREQ), which uses action research and applied analysis methodology of thematic analysis in its stages as a theoretical foundation^(17-18)^. The analysis construction is anchored in the perspective of preparing a report using the same methodology, which was later reviewed together with all records under the theoretical umbrella of the bioethical perspective.

### Methodological procedures

Data collection took place during the thematic seminar event, through analysis of forms and recording of them, with interventions from participants. It was divided into three moments:

- First moment: begins with a questionnaire accessed by participants on Google Forms^®^, containing five questions, namely: full name; email; in cases of public health emergencies, is it right for the right to confidentiality and anonymity to be restricted? (Justify your answer); how does tracking people violate individual freedoms?; would you install an app on your mobile device that would allow to map your movements? (Justify your answer);
- Second moment: thematic seminar presentation, with recording of the content of the discussions on Google Meet^®^, guided by bibliography that considers ethical implications of tracking devices related to benefits and harms, intelligent physical distancing, conflicts between freedom and privacy, mandatory use of applications and institutional and professional responsibilities^(16)^;
- Third moment: repeating the questionnaire after the discussions with the same questions answered before starting work.

After transcribing the speeches and interventions during the seminar, the material was separated and cataloged to systematize the analysis.

### Study setting

The research was carried out in a *stricto sensu* graduate program at a public university in the state of Minas Gerais.

### Data collection and organization

The study took place during a thematic seminar presentation in the discipline of Bioethics, using Google Meet^®^ to record the discussion and Google Forms^®^ to collect participants’ responses on the topic in the first and third moments.

Participants were professionals from nursing, medicine, dentistry and nutrition and accounting sciences with health auditing, in a focus group held in a single meeting. Therefore, non-probabilistic criteria were used, occurring for convenience or access^(19)^. Inclusion criteria included being enrolled in a bioethics program and discipline or being a bioethics professor in the first semester of 2021 and having participated in the class on bioethical conflicts regarding individual tracking device use.

Data were collected as follows: the first moment involves answering the questionnaire on the platform; the second moment comprises the dialogued exposition of the topic in the virtual environment; and the third moment comprises new collection of information, also on a platform.

### Data analysis

The data collected followed pre-analysis stages with reading immersion, material exploration, coding and processing of results obtained^(18)^. The information contained took into account: a) semantic cohesion; b) creation of codes, considering the characteristics of the most relevant data and conceptual definition of each code; c) topic research, grouping codes into emerged topics and review of topics by evaluators and thematic map preparation; d) definition of topics under agreement for each topic separately; e) report preparation with selection of examples of excerpts from participations and observations. Listed issues were categorized in the analysis, such as individual freedom and collective responsibility, right to confidentiality and anonymity, spontaneous demand, restrictions on data availability, duration of intervention effects, implications for professional health practices. These were summarized into three thematic axes, such as the right to secrecy and anonymity, individual freedoms and personal choice and spontaneous demand for individual tracking applications.

## RESULTS

As a systematization of the topics identified from thematic analysis of collected material, records were made in order to contemplate the relationships between the discussions at the three moments. Coded elements relate to participants’ personal and professional positions, based on the proposed intervention. The results presented follow the logic established from the discussion pattern.

### Personality in secrecy, anonymity and individual freedoms for the “common good”

At first, participants presented arguments that assume that concerns are about understanding the issue from a personal perspective. When asked about the restriction of the right to confidentiality and anonymity in specific cases of health emergencies, all participants agreed. First-person terminology and statements that refer to codes such as “common good”, “collective interest”, “collaborate”, “understand” and “everyone” were used.

*Yes, I think that, when using the application on my cell phone, I would have the benefit of knowing if I had contact with someone infected. Nowadays, we are all on the streets without knowing it. It could be anywhere. So, this is what the collective interest is: helping each other.* (P3 - First moment)

*Yes, I wouldn’t see a problem. It’s an attitude for the common good, as long as everyone uses it. This way, COVID goes away faster.* (P4 - First moment)

*Yes, if the situation of collective need requires actions that determine disease prevention or control, for instance.* (P8 - First moment)

Regarding individual freedom rights, participants suggest, in principle, that each person’s rights be observed. However, they bring discussions about the need for collective collaboration in emergency cases. The same can be said when asked about their willingness to install a contact tracing app on their cell phone:

*Each person makes their choice. Yes, I would install it. It would be good for me and good for everyone.* (P2 - First moment)

*I agree to use. To control an illness like COVID, it’s worth the effort. This is a time when we need to express solidarity.* (P5 - First moment)

*In a global understanding, I think it’s worth it. It would help to identify where the disease in question is being transmitted so that we can intervene.* (P8 - First moment)

### Debate at seminar: professional aspect of the perspective of intervention in personal life

During a seminar, reports begin to alternate while data involving the General Data Protection Law is presented^(13)^. There is a rupture in the discussion between the personal and academic debate that immediately points to issues that concern professional practice. The reluctance to give up something for the common good migrates to challenging the current security of using patient data and other experiences, such as electronic medical records:

*I have already worked with data from patients with more serious illnesses. The use of SINAN, the report to the centers with the person’s name, I always understood as safe. Now, observing the person on the cell phone, I have a lot of reservations. You don’t need to go that far.* (P3 - Second moment)

*That you can preserve patient data only for people who are there. When we send our notification forms to health surveillance, I am, in a way, breaking the confidentiality of a appointment. They send there for surveillance for a person who was not at that appointment.* (P5 - Second moment)

*I think that, even in cases of public health emergencies, we must maintain confidentiality and anonymity to comply with ethical precepts related to human beings, right?* (P6 - Second moment)

*I already said that I wouldn’t install it* [...] *I still have the same opinion. Until they prove to me that it works one hundred percent and that everyone installing it makes a difference in the pandemic, I won’t change my mind, nor will I recommend it to my patients.* (P7 - Second moment)

In the third moment, the repetition of questions shows a reversal of trend with a deepening of conditions that were previously not even mentioned by the group. Topics arose such as requiring data to be used for a period of time until it is deleted, mentions of other situations in which data protection has been breached due to hacker invasions. The fear of breaking the relationship between professional, healthcare team and user, in case of failure of the method, is contained in the statements that now impose conditions of a technical nature or simply reject the initiative.

*No, the information about where I have been can even be used to stalk me. The fragility of data use must be taken into account, since nowadays there are many cases of leaks that expose people.* (P2 - Third moment)

*No, because I don’t want information obtained from my phone to be available without me knowing what it will be used for. I fear for myself and my patients.* (P4 - Third moment)

*In public health emergency situations, it is advisable to adopt measures to track sick people or contacts so that it is possible to block and reduce the transmission of disease-causing agents with the potential for significant morbidity/mortality. This is even a practice recommended by the WHO and applied very early on by humanity to control epidemics. Confidentiality is somewhat broken through compulsory notifications; however, despite this breach, the security of a person’s data must be maintained through surveillance bodies, bearing in mind that patients’ right to non-stigmatization and non-incrimination. I don’t think personal data should be made available without knowing how it will actually be used.* (P5 - Third moment)

*It’s a reality of no return, but I don’t think I would be safe in that. Some countries will force people to use it, which is wrong.* (P7 - Third moment)

## DISCUSSION

### Right to confidentiality and anonymity

In cases of public health emergency, is it right for the right to confidentiality and anonymity to be restricted? In the analysis of the content collected in the first moment, an emphasis on answers regarding confidentiality is noticeable, not being repeated when referring to the “anonymity” present in the first question. There is a breakdown of assertions on the topic. Participants agree that it is possible to allow an invasion for the good of the community in a personalistic way and without imposing conditions. Secrecy and anonymity deal with bioethics on the premise of non-negotiable rights that support individuals’ autonomy^(16)^.

The exceptions previously mentioned would be regulation based on state power, in order to ensure that the information is used for scientific purposes only. In the Charter of Rights of Health Users, secrecy and anonymity are understood as conditions of protection for service users, remembering that their actions must provide for the protection of these two premises in all stages of the care or assistance process^(20)^. Analysis conducted by Pagliari^(21)^ points out that major issues related to the topic are precisely about confidentiality, even though tracking individuals for monitoring is a practice considered usual in the health area. Anonymity, data security and the protection of individual rights that are typical of free societies are considered key elements.

As a conclusion to the discussions related to the topic, there were several mentions in the third moment of the sedimentation of collective understanding, of the notion of delimiting interference in individual rights in tracking technology use. Quotes involved terms such as “collective good”, “good for all” and “safety”.

During the second moment, the condition of secrecy and anonymity is fundamental for tracking people, as exemplified in P5 and P6. The fundamental argument is that the number of people who do not mind giving up their privacy in some way for the common good is much greater than the number of people who do care. Thus, as long as an agreement based on the democracy of nations and with well-defined rules is preserved, the strategy would be accepted^(16)^. Therefore, after approaching the questionnaire in the third moment, the need to adapt conditions arises, preserving the ethics of autonomy.

Recent studies indicate that the method of tracking individuals does not demonstrate certainty about its effectiveness, considering that the conditions highlighted in the research are related and there is no 100% safe initiative^(5)^. The most popular applications in Europe and North America store user data centrally, which would make them vulnerable to hacker attacks when stored on an individual’s mobile device^(22)^.

Recently, the Federal Supreme Court (FSC) reaffirmed in an extraordinary appeal the citizen’s right to their personal and health data, in a case where the Federal Public Ministry requested the Federal Council of Medicine to issue a resolution authorizing doctors, managers and institutions to making medical records and other information available without authorization from patients or their families^(23)^.

### Of individual freedoms and personality of choice

In addition to reflecting on the individual right to confidentiality and anonymity of personal information, it is correct to separate this reflection from another, equally important, but within the scope of the right to come and go: the issue of freedom. The COVID-19 pandemic had as a major global consequence the restriction of people’s commuting. In a world where we have ease of intercontinental travel, it is necessary to analyze how long it is a state right to restrict individual freedom.

Such debate today is in the personal and collective dimensions, encouraged bellicosely by political-partisan positions. As the second question in the questionnaire, participants were asked: how does tracking people violate individual freedoms?

Blocking travel dramatically affects the right to come and go. The central idea discussed during the seminar is the logic of the so-called relative benefit. Freedom restriction is seen as an incarceration that people want to get rid of as quickly as possible. Given this, the acceptance of a tool that restricts displacement for a short period of time, with a glimpse of later release, is well accepted by the majority, as in P4, P5 and P8. The view would therefore clash with the restrictive power of the state in prison, or conditions associated with crime.

In a debate about social and collective commitment, there are doubts about the directions taken in Brazil that may violate basic principles of principlist ethics. For participants, the limitation of individual freedoms supports the perception that there must be individual security, as mentioned by P6 and P7, and must be promoted by the State. It is a reflection of trends in the pandemic of limitations in discussions about invasion of privacy during the pandemic in the United States, with the active search carried out with contacts after the first detection of proximity to a case of COVID-19^(24)^. Governments in countries such as China, South Korea, Israel and India adopted extremely coercive strategies to combat COVID-19^(21)^. Subtle bioethical questions support the position adopted after the seminar. In totalitarian regimes, there is an obligation, and in established democracies, judicialization of bioethics to guide decisions^(24)^. In particular in South Korea, devices were used to “precisely track the location of identifiable individuals, personal data mining, comprehensive facial recognition technology, and body-contact devices to enforce quarantine”^(25)^. The following category reinforces the perceived need for personal control or data security.

### Spontaneous demand for individual tracking apps

According to studies by Parker^(16)^, the particular experience of making oneself available to the state to disseminate information of a personal nature is relative, and is also a price to be paid only with clear rules and under the pretext that it brings some relief or security later. The question is: would you install an application on your cell phone that would allow to map your movements? The provocation contained in the first and third moments provides manifestations of initial agreement and subsequent denial due to bioethical considerations related to autonomy, non-maleficence, justice and beneficence in the health relationship as a service provider.

In the discussions encouraged by the subject, it is possible to see a repetition of terms that are associated with assertiveness for the question. Only P7 refuses to register a monitoring device at the first moment. Again, the most frequently presented codes are “blocking”, “objective”, “preservation”, “ethical principles” and “benefits”, in an antagonistic representation of responses.

Although they seem to be divergent codes, there is greater convergence with the other participants’ reports with the assertion of allowing control, as long as it is possible to interrupt it according to their will. During the second moment, issues are raised about the economics of technology in health and the social strength to implement innovations. It is important to mention the discussions about the personal understanding of the rights of healthcare users in Brazil and also their implications for telemedicine and other tools. Safeguarding the professional relationship and preserving users’ right of access, in addition to developing its own legislation, is a pressing need^(26)^.

Research participants in the third moment assume that there is individual discretion that overrides collective well-being. As previously considered, in countries with closer political relations, citizens were conditioned to comply with determined strategies, whether through the imposition of fines, unemployment or imprisonment^(27)^.

In the final discussions, the post-seminar questionnaire analysis shows a predominance of assertive codes, including the terms “prior agreement”, “data protection”, “collective good” and “rights”. The words “individuality” and “right” appear mentioned in participants’ denials, and these are the same ones who declared that they did not accept the installation of tracking applications in the first moment questionnaire.

Recent studies analyzing the legal legitimacy of use of these technologies in the light of bioethics highlight the need to limit the time of use of applications and eliminate data. According to Ram and Grey^(28)^, it is precisely the lack of defined time to close the monitoring of the said epidemiological, which allows data appropriation by third parties, hacker invasions and various uses for purposes other than those previously authorized.

Morley *et al*.^(29)^ consider that the problem needs to answer questions that were listed by study participants: procedure duration; its effectiveness; participation voluntariness; the purpose of data collection; and the availability time of this data. The prospect that data can be deleted according to individuals’ wishes is taken into account, which would be called “decommissioning”.

Research participants evoke professional action not to support the new technological method proposed as a matter of urgency, but as if they considered previous methods to be gentler and less invasive than tracking devices.

In the third moment, the support of a position of distrust based on preservation of freedoms and rights of patients contradicts the majority’s initial response: not installing the monitoring application and not recommending it, said by P2, P3, P4, P5, and P7. The approach refers to the practical use of utilitarian and principlist ethics. In the first, by protecting principles that are not related to a pre-established logic, but rather shaped case by case based on individual morals and personal experiences. This is the case when codes show that professionals care about exhibition, thinking first of themselves, of their civic action, and then creating a professional interface.

Utilitarian ethics appears in the maximization of well-being based on the feeling of universal justice. When the mentions in the seminar give the feeling of imposed initiative, of governmental authority, participants opt for repression in constructions that permeate professional values of bioethics, such as patient rights and human rights^(30-32)^.

The categories presented demonstrate that students have a propensity for collective collaboration in the face of the pandemic. During the second moment and with the culmination of the third, professional issues, such as security of data from medical records and other systems already used, conceived as “not safe”, cause them to present reservations about using devices from a professional perspective as well.

### Study limitations

Although the study’s object of work is a focus group as a way of identifying perceptions in an area that is under open discussion, it is necessary to expand the discussion to other groups, with social actors also directly impacted. These perceptions must be analyzed not only in professional groups that are part of highly qualified educational programs, but also taking into account the diversity of our society.

### Contributions to nursing, health, or public policies

The study demonstrates that the acceleration of changes in the practice of using tracking devices in healthcare, caused by the COVID-19 pandemic, raises profound bioethical issues of privacy, individual rights and state-individual relations. Confronting these questions with participants deepened the discussion according to the reality experienced, which was based on concerns about the professional mission dynamics. It demonstrates the need for constant debate on health tracking technology device use, passing the critical period of the pandemic so that the best conduct is taken and technology use is always in favor of safety and well-being of patients, users, professionals, service managers and society as a whole.

## FINAL CONSIDERATIONS

Individual tracking technologies were considered an alternative for preventing COVID-19, and as soon as the world was faced with out-of-control transmission and the lack of more effective means of contact. The application of technology that monitors individuals or restricts individual rights is viewed with suspicion, even when considering the supposed benefits of its mass use. When faced with this new paradigm in the list of actions specific to the pandemic and a subsequent extension of the practice to other activities in public health and epidemiology, there is a certain insecurity motivated by values linked to bioethics both in the personal and professional spheres.

From the analysis of the material produced by the seminar, it was evident that there was concern about the consequences of using individual tracking technologies by participants after presenting an argument on the topic, giving rise to leading role and personal autonomy as well as control of the state in practice for health work. This way, participants move from their personal opinion on the topic to reflection on the implications for their professional performance in health.

COVID-19 was not the first pandemic and will probably not be the last experienced. Using this type of device in this context brought lessons that could be used in other situations of equal or greater severity, without major obstacles. Its use should be encouraged, as long as confidentiality, secrecy and freedom of choice are also ensured and respected. This reality demonstrates the need to develop new mechanisms to preserve these precepts in the use of tracking devices.

## Supplementary Material







## Data Availability

https://doi.org/10.48331/scielodata.CGLEQU
