# Supplementary material for: The use of individual tracking programs in public health: a bioethics dilemma
Source: Rev Bras Enferm. 2024 Aug 19;77(Suppl 4):e20230041. doi: 10.1590/0034-7167-2023-0041 (PMC11338528; doi:10.1590/0034-7167-2023-0041)
Supplement: Supplementary file 1 [file 0034-7167-reben-77-s4-e20230041-suppl01.pdf]

**Uso de tecnologias de rastreamento de indivíduos**		
**Percepções individuais Pré-seminário**		
**Questões**	**Relação entre as respostas**	**Análise temática**
1. Em caso de emergência de saúde pública, é correto que o direito ao sigilo e anonimato sejam restringidos?	- Interrupção do direito ao sigilo e anonimato em casos de risco coletivo pelo bem comum;<br><br>- Justificativa de que já existem casos em que a quebra de sigilo pessoal e anonimato são interrompidos por questões legais.<br><br>- Concordância, desde que em situações em que vidas sejam salvas.	- A maioria dos participantes concorda, e faz ponderações a respeito da iniciativa pessoal de cada um. Citam-se termos como “bem comum”, “interesse coletivo”, “ajuda”.
2. De que forma o rastreamento fere liberdades individuais?	- É preciso ponderar em que situações o direito individual se sobrepõe ao coletivo; Existem situações em que há a necessidade de se impor o bem comum como meta.<br><br>- Preservação da dignidade humana	- Os participantes manifestam-se na perspectiva de uma intervenção global, em que eles estejam colaborando para a resolução de um grande problema. Citam-se termos como: “universal”,

		‘colaboração”, “necessitados”.
3. Você implantaria em seu dispositivo móvel um aplicativo que permitisse que mapeassem seus deslocamentos?	- Concordância geral sobre a necessidade em situações extremas.<br><br>- Preocupação com o bem coletivo	- Preocupação manifesta dos participantes com o bem coletivo na perspectiva pessoal. Visão pessoal do uso do dispositivo como mecanismo de solidariedade.<br><br>-Não são apontadas restrições de caráter técnico, como armazenamento e risco de invasão dos dados para outras finalidades.<br><br>“tratar”, “diagnóstico”, “uso específico”.
**Percepções individuais Pós-seminário**		
**Questões**	**Relação entre as respostas**	**Análise temática**

1. Em caso de emergência de saúde pública, é correto que o direito ao sigilo e anonimato sejam restringidos?	- Discordância do uso obrigatório;<br><br>- Entendimento de que já existem situações em que essa imposição acontece.<br><br>- Citações de experiências profissionais em que dados dos pacientes ficam expostos, direta ou indiretamente.	- Posicionamento profissional de que o uso coletivo é necessário, mas não deve ser imposto;<br><br>- Percepção de que uma evolução no uso de dados pessoais na relação profissional pode significar uma perda de controle. “Ataques”, “hackers”, “invasão”
2. De que forma o rastreamento fere liberdades individuais?	- Os aplicativos podem coletar informações em excesso.<br><br>- Risco de extravio de dados.<br><br>- Necessidade de rigor ético.	- Preocupação profissional com outras formas de acesso a dados do paciente, como prontuário eletrônico e sistema de notificações.<br><br>- Necessidade de garantias legais sobre os dados, por receio de extravio destes.<br><br>“segurança”, “insegurança”, “direito”.

3. Você implantaria em seu dispositivo móvel um aplicativo que permitisse que mapeassem seus deslocamentos?	Imposição de condições que garantam segurança, individual e coletiva. Todos os participantes demonstram preocupação com controle dos dados e duração da intervenção.<br><br>- Manifestações de preocupação de exposição de pacientes, na condição de exercício profissional	- Os participantes concordam, mas a maioria passa a exigir controle sobre a duração dos eventos e tempo para eliminação definitiva dos dados coletados. Sugerem “controle”, autonomia”, “tempo de uso”, “proteção”.
