# Supplementary material for: The use of individual tracking programs in public health: a bioethics dilemma
Source: Rev Bras Enferm. 2024 Aug 19;77(Suppl 4):e20230041. doi: 10.1590/0034-7167-2023-0041 (PMC11338528; doi:10.1590/0034-7167-2023-0041)
Supplement: Supplementary file 2 [file 0034-7167-reben-77-s4-e20230041-suppl02.pdf]

SEMINÁRIO BIOÉTICA – PARTICIPAÇÕES EM AULA

TECNOLOGIAS DE MAPEAMENTO ATRAVÉS DE DISPOSITIVOS MÓVEIS

TRANSCRIÇÃO DOS ÁUDIOS

RESPOSTAS DOS QUESTIONÁRIOS PRÉ-TESTE

Questões:

Questão 1. Em casos de emergência de saúde pública, é correto que o direito ao sigilo e ao anonimato sejam restringidos? (Justifique sua resposta)

Questão 2. De que forma o rastreamento de pessoas fere liberdades individuais?

Questão 3. Você instalaria em seu dispositivo móvel um aplicativo que permitisse que mapeassem seus deslocamentos? (Justifique sua resposta)

E1

1. Existe exceção nestes casos. Apesar disso todo mundo precisa entender que tem o bem comum em jogo.
2. As pessoas tem direito a liberdade e neste caso pode ferir no sentido de impedi-la de ir e vir ou de agir.
3. Sim. O momento pede.

E2

1. Sigilo sim. Porém, meios precisam ser encontrados para reduzir os malefícios coletivos assegurando os direitos individuais.Mas vale pelo bem comum.
2. Concordo. Cada pessoa faz sua escolha. Eu instalaria sim. Seria bom pra mim e bom para todos.
3. Sim. Desde que fosse possivel o bloqueio transitorio conforme o meu desejo e contratos previamente assumidos.

E3

1. Eu penso que para o uso de aplicativo no celular eu teria o benefício de saber se tive contato com alguém contaminado. Hoje em dia estamos todos nas ruas sem saber. Pode estar em qualquer lugar. Então, o interesse coletivo é isso: Uns ajudando os outros

2. O rastreamento pode constranger as pessoas, tirar das pessoas em alguns momentos sua autonomia.
3. sim, porém ficaria receoso sobre minhas informações pessoais.

E4

1. sim, o bem coletivo justifica essa restrição. Não veria problema. É uma atitude pelo bem comum, desde que todo mundo use. Assim a covid vai embora mais rápido. Temos que colaborar.
2. quando fere o princípio da dignidade humana e viola direitos fundamentais
3. sim, a depender da justificativa objetivando bem coletivo

E5

1. Apesar de o direito sigilo ser uma premissa fundamental em várias atividades que envolvem a intimidade de uma pessoa, há situações em que o mesmo pode ser quebrado. Temos, como exemplo, o sigilo bancário e telefônico em caso de crimes e, no campo da saúde, a quebra do sigilo sobre condições clínicas quando há risco de vida para terceiros ou mesmo para a pessoa (desde que essa não possua a capacidade de decidir-se sobre a sua saúde de maneira adequada). Em caso de emergências em saúde, esse direito poderia ser quebrado no caso de risco para terceiros, como o que foi observado na pandemia de COVID-19. Todos tem que ajudar.
2. Rastrear pessoas pelo mero intuito de o fazê-lo sem observação de preceitos éticos pode ferir o direito à intimidade, configurando-se um invasão de privacidade o que fere direitos individuais fundamentais. Porém, esse poderia ser um recurso viável para monitorar situações de saúde pública, como observado na situação vivenciada pelo mundo durante a pandemia de COVID-19. Há algumas experiências exitosas de países que utilizaram a tecnologia de rastreamento no enfrentamento da disseminação do vírus SARS-COV 2. Eu concordo em utilizar. Para controlar um mal como a covid, vale o esforço. É um momento em que precisamos expressar solidariedade.
3. Permitiria desde que o mesmo tivesse um objetivo claro e seguisse devidamente princípios éticos. Não gostaria de ficar exposta.

E6

1. Sim. É o interesse de todos... em se tratando do problema atual, é necessário. Eu apenas temo pela exposição pessoal.
2. A medida que você divulga as informações de forma universal.
3. Não, porque quero privacidade sobre a minha vida.

E7

1. A liberdade da manifestação do pensamento, inclusive garantindo-se o anonimato. Pensando em todos, sempre.
2. Direitos humanos garantem a todos o direito mais elevado nivel possível de saúde e obrigam os governos a adotarem medidas para evitar ameaças a saúde publica e prestarem assistência medica a aqueles que necessitam.
3. Não instalaria. Temo pela minha privacidade.

E8

1. Sim. Se a situação da necessidade coletiva exigir a ações que determinem a prevenção ou controle de doenças, por exemplo.
2. A partir, do momento que o individuo é limitado em suas escolhas, quando o rastreamento tem algum impacto nos modos de vida, ou provoca intervenções de terceiros. Num entendimento global, acho que vale a pena. Ajudaria a identificar onde está se transmitindo a doença em questão para intervirmos.
3. Sim. Como afirmei, é necessário colaborar.

SEMINÁRIO BIOÉTICA – TRANSCRIÇÃO DE PARTICIPAÇÕES EM AULA

TECNOLOGIAS DE MAPEAMENTO ATRAVÉS DE DISPOSITIVOS MÓVEIS

0:03 - 0:37

Apresentador 1 Para pessoal eu e Apresentador 2, a gente se esforçou em fazer uma discussão que siga o escopo com que os colegas utilizaram também mas que a gente possa trazer também algo de novo para todo mundo com base no artigo que nós utilizamos para orientar essa discussão. Nós vamos falar um pouco sobre tecnologia de rastreamento. Nós vamos falar um pouco sobre o monitoramento de pessoas e de seres humanos sobre o direito de ir e vir, além do deslocamento das pessoas no planeta de uma maneira macro.

• 0:37 - 1:08
Apresentador 1: Depois a gente sabe também para ouvir de vocês experiências particulares específicas de cada um e inevitavelmente como o artigo trata, nós vamos falar de questões relacionadas à pandemia com o vídeo porque também é o assunto em voga no mundo inteiro. Não tem como a gente fugir disso. E isso abalou, interferiu e modificou de alguma forma o direito de ir e vir das pessoas. Para começar, Apresentador 2... Se se quiser dar um bom dia pessoal pode ficar à vontade.

• 1:08 - 1:38

Apresentador 2: Enquanto você foi pegar a água eu dei bom dia pro pessoal... Quero também registrar a todos que foi um prazer trabalhar com o colega Apresentador 1. Nós podemos fazer várias meets durante esses dias para podermos alinhar nossa discussão e trazer conteúdo para esse dia. Obrigada pela oportunidade de trabalhar com você e o professor, pôde nos oportunizar esse momento.

• 1:39 - 1:43
APRESENTADOR 2: Espero que tenhamos aí uma excelente manhã e produtiva.

• 1:45 - 2:20
Eu comentei com a APRESENTADOR 2 e acho que eu não cheguei a comentar aqui na disciplina. Acho que nem com professores meu orientador que eu leciono bioética. Só que eu percebi que eu sou um amador. Eu tenho aprendido muito muito muito, tenho mudado muitos conceitos aqui. Em princípio nós vamos começar aí, vamos dar uns 10 minutos, 10 a 12 minutos, para vocês preencherem esse formulário aí e através desse formulário que a gente vai orientar as nossas discussões.

• 2:20 - 2:36
E são perguntas muito simples mas eu gostaria que vocês,, se possível se isso esforçassem ao máximo na argumentação para pra gente poder ter um material bacana para discutir no final. Então vou contar dez minutos aqui a gente retorna a vou mandar lá no WhatsApp também.

• 0:00 - 0:00

• 12:15 - 12:22
Quantos ainda para responder quem está monitorando Apresentador 2 sou eu.

• 12:22 - 12:25
Até agora três três responderam.

• 12:29 - 12:30
Mais 13 minutos.

• 12:33 - 12:35
Faltando dois minutos para completar os dez.

• 0:00 - 0:00

• 15:55 - 16:16
Apresentador 1: Já temos seis pessoas que responderam aqui. Só faltaram duas pessoas. Daremos mais tempo.

• 16:25 - 16:26
Apresentador 1:Faltando uma pessoa.

• 17:44 - 18:08
Apresentador 1: Agora responderam todos. Nós podemos começar, não tem mais ninguém respondendo, acho que não. Não posso deixar de encerrar aqui. Então pessoal, é isso. Deixa eu fazer a projeção.

• 18:43 - 19:14
Vamos começar então: as nossas reflexões são baseadas na lógica de um conflito bioético que exista a partir do uso de tecnologias de rastreamento de indivíduos. Esse tipo de conflito, ele teria num primeiro momento (e ao se fazer uma primeira leitura inicial), teria surgido a partir da disponibilização em larga escala de dispositivos móveis. Eu vi que é pequena a tela compartilhada por inteiro.

• 19:15 - 19:53

Agora melhorou. Pra mim tá bom aqui tá bom. Agora sim. Sentirá melhorada hilárias. Não não aqui que vocês estão conseguindo ver até lá na apresentação. Agora a gente começa, pessoal. Num primeiro momento, aparentemente, essas tecnologias elas que teriam fomentado esse problema de rastreamento de pessoas.

• 19:53 - 20:37
No entanto quando a gente começou a fazer as nossas leituras e a gente é sempre interessante é importante que a gente faça uma revisitação histórica dos porquês das coisas a gente encontrou um problema que precede a tecnologia que capacita para rastreamento de pessoas. Na verdade o deslocamento humano ele é o grande causador de conflito, provavelmente um grande causador de encontros também, mas também o grande causador de conflito ao longo da história. Aqui nós isolamos alguns fatos que nós julgamos que fossem importantes para que nós fizéssemos uma pré- reflexão a partir do uso de tecnologias de rastreamento.

• 20:37 - 21:07
Então eu vou falar um pouco aqui num panorama respeito dessa reflexão prévia. Depois nós vamos assistir alguns vídeos que estão relacionados mais à questão atual do uso de tecnologia de rastreamento de indivíduos; são quatro vídeos e aí a gente abre depois para as colocações dos colegas a partir de questões que elencamos como importantes para essa discussão na etapa final. A gente faz então uma apresentação do que diz o artigo que nós escolhemos como norteador da proposta de discussão.

• 21:08 - 21:44
Então tá. A origem do controle de deslocamento de indivíduos, a princípio pelo menos no que é o que a gente encontra nas literaturas mais antigas, diz que a prática nômade das culturas humanas, por se tratar de grupos humanos que tinham e inúmeras divergências inclusive fenotípica entre si e ao se encontrarem na prática de moradia temporária em determinados locais por conta de utilização até a escassez dos recursos de determinada localidade, Eles entravam em conflito. Por conta de um de uma luta ali, uma briga por espaço. Essa briga por espaço, ela resultou, segundo a maioria dos

historiadores dos antropólogos da área, resultou na extinção de muitas espécies as quais nós não chegamos a formar inclusive entendimento sobre elas e onde elas se localizam entre os grandes grupos que nós temos como estruturas de estudo: seriam os Australopithecus fica e vir até os os neandertais, o Homo erectus, o Homo habilis lá atrás, até chegarmos no Homo sapiens como nós conseguimos conceber como ser humano mais próximo do que nós temos hoje em dia. E isso vem se arrastando por muito tempo.

• 22:36 - 23:15
Chegou um momento em que quando o ser humano,, isso há mais de 30 mil anos atrás quando o ser humano ele consegue se estabelecer em um local fixo de residência a partir do domínio do fogo, da agricultura e da caça, Esse ser humano ele começa a formar unidades de liderança. Ele começa a colocar indivíduos, unindo o grupos em torno de uma referência. Embora isso talvez já existisse tempos atrás, lógica que nós temos hoje é imaginar um líder como um indivíduo mais velho. Essa regra não era aplicada em todas as civilizações. Pelo contrário! As pessoas mais velhas eram por vezes deixadas pelo caminho.

• 23:15 - 23:58
Entendia-se que elas não tinham grande utilidade para as tribos e isso, esse tipo de conduta, se arrastou até os vikings, que já não tem mais tanto tempo assim. A gente colocar isso escala. Os movimentos escravagistas que ganham uma grande amplitude e principalmente com a ascensão a partir da expansão do Império Egípcio. Os grandes escravagistas eles tinham por prática captura de pessoas que estivessem em deslocamentos de áreas de uma área para a outra. Eram consideradas apátridas e escravizados para aquele povo específico.

• 23:58 - 24:34
Um grande fato citado no Antigo Testamento da Bíblia foi a liderança de Moisés de tirar o povo hebreu do Egito através de um grande processo migratório que culminou em uma história fantástica. Da Bíblia, todo mundo conhece. esse deslocamento, essa migração de uma área para a outra significava para alguns a escravidão e para outros a liberdade do processo escravagista.

• 24:35 - 25:18
A ideia de ocupação de territórios ainda habitados e não colonizados e deslocamentos para locais inóspitos nos vem provavelmente das grandes navegações. No entanto elas não surgiram com as Grandes Navegações. A lógica de achar que os portugueses chegaram ao Brasil e encontraram um local e desabitado. Por isso eles descobriram o Brasil era uma terra previamente habitada. Inclusive nos livros de história. Hoje já está provado que inclusive existiam rotas muito mais antigas que a influência dos portugueses em 1500, de civilizações estruturadas que se deslocavam inclusive dentro da região amazônica.

Nem dentro da selva existem rotas lá onde eles se deslocam subiam até próximo da América Central para estabelecer negócios, entre outras atividades. A definição e proteção de fronteiras entre países é um procedimento que vem... ele não é tão antigo

assim... Até procurei uma arte para poder mostrar, porque eu achei interessante a gente buscar pelo menos de onde vem a lógica do passaporte, né?

• 25:55 - 27:28
A primeira referência de passaporte é feita em 450 antes de Cristo. Aqui foi um oficial servidor do rei Hartaxerxes I da Pérsia, que pediu permissão para ele ajudar, e o rei concordou e deu a ele uma carta destinada aos governantes da província do até lá do Rio, requisitando para ele uma segurança enquanto ele estivesse em terras estrangeiras não é essa. Essa história seria o primeiro registro e a lógica da palavra passaporte vem da França há já muito tempo depois no século 14, em plena pandemia de peste negra que praticamente dizimou a Europa inteira, matando aldeias com uma epidemia de peste bubônica associada a relações de higiene que povos não estabeleceu... e a gente vai falar um pouco mais sobre isso, onde essas questões de saúde coletiva elas entram na discussão. O rei e determinou a seus favorecidos, entregava as cartas que lhes davam passagem pelos portos e elas eram intituladas pass port, que literalmente significa passar de um porto ao outro no idioma francês . A palavra passaporte então é constituída e tem algumas pessoas que falam que esse termo vem da Idade Média mas era também a mesma lógica: de que autoridades locais tivesse o direito de passar por determinadas áreas sem serem incomodados.

• 27:30 - 28:04
Existe na internet inclusive se alguém tiver curiosidade de ver existem algumas imagens interessantes e passaportes muito antigos, de 1800 e alguma coisa. Já eram utilizados em forma de um caderno como muitos países adotam até hoje como convenção internacional. Então nós a definição e a proteção de fronteiras entre países como um evento novo e a própria lógica da migração o que é um princípio desde a percepção da migração nômade.

• 28:04 - 28:36
Depois a migração domina ações absolutistas como se ter um império egípcio e outros períodos na Idade Média. a gente tem a partir do sistema feudal, da delimitação dos feudos da pessoa entradas no feudo depois entrar na cidade. E como na Revolução Industrial na Europa e depois mais tarde aqui no Brasil até bem recentemente, a gente tinha os movimentos migratórios dentro dos países, que estão entre as mais diversas busca de trabalho que todo mundo conhece né. A história do pessoal que saiu do Nordeste fugindo da seca e se dirigia a São Paulo em busca de trabalho, em busca de melhoria de vida. E todos esses movimentos de êxodo e de migrações são dignos de estudo, inclusive de revisitação constante até hoje para poder entender o comportamento dinâmico da sociedade. Então a gente tem aí também esse controle e o estabelecimento de um controle internacional de fluxo de pessoas através de um registro formal, que são os passaporte.

• 29:09 - 29:54
E aí a para fins de controle epidemiológico e sanitário que é uma parte que interessa muito a nós, a gente tem um controle de deslocamento de indivíduos e a partir de uma doença que assustava todo mundo que era hanseníase. Dizem que a hanseníase ela chegou à Europa pelos movimentos expansionistas dos persas. Tem outros autores que

consideram que não foi isso que aconteceu, mas com o advento da hanseníase, tão citada no Antigo Testamento, no Novo Testamento, bem como registros mais antigos... A lepra causadora de estigma e de pavor de parte das pessoas impunham a um indivíduo doente.

• 29:54 - 30:31
E aí entra um foco importante para a nossa discussão: a segregação social. Ele abandonar aquela área de ocupação por força do Estado, por força do poder absoluto. Ele abandonar aquela área para viver em outro lugar. A lógica do leprosário é muito antiga inclusive, porque o banimento que era inclusive conduzido por um padre: era feito uma missa de corpo presente do chamado leproso como se ele tivesse morrido a partir daquele momento por ter uma doença incurável e transmissível.

Ele era banido para morar em regiões cavernosa longe das cidades, sem nunca mais poder voltar, inclusive sem poder ocupar espaço nas estradas. E o leproso inclusive poderia ser punido com a morte caso fosse capturado, se encontrado numa estrada ou local de trânsito de pessoas. Para tentar evitar (e aí se arrastou também por muito tempo) a hanseníase carregou a lógica do estigma. Até a década de 70, se não me engano a proposição do nome, de nome da mudança de nomenclatura de lepra para hanseníase.

Mas isso ainda existia aí e foi mais ou menos diminuindo com o advento da polioquimioterapia, que é utilizada hoje para tratamento da doença. Os primeiros registros do banimento estão lá no Levítico: “ todo o homem é atingido pela lepra terá suas vestes rasgadas e a cabeça descoberta cobrirá a barba e clamará: impuro! Impuro1 enquanto durar o seu mal ele será impuro e impuro Enquanto durar o seu mal, habitará só, e sua habitação será fora dos acampamentos.

• 31:44 - 32:19
Numa lógica de exílio forçado. E nesse caso aí conduzido pela Igreja porque o profissional sacerdote era o mais indicado, mais recomendado. inclusive nos textos do Levítico, nesse mesmo capítulo aí, falam de várias orientações para a avaliação das lesões a avaliação das manchas. Partindo do sacerdote atuando ali talvez como uma figura primária à questão do médico como nós conhecemos.

• 32:21 - 32:53
E aí a gente tem algumas algumas questões que já citei, como controle de deslocamento de pessoas da Idade Média, e no Brasil isso vem a ser instituído de alguma forma com a vinda da família real pra cá, nós passamos um hiato de mil e quinhentos aproximadamente, até 1808, com o deslocamento da família real para o Brasil fugindo de Napoleão Bonaparte. Nós passamos por um período onde o Brasil não tinha o estabelecimento de leis sanitárias.

• 32:54 - 33:49
O primeiro esboço foi feito aí um ano antes da ida, do retorno da corte da família real para Portugal. Eles montaram alguns hospitais de Salvador até o Rio de Janeiro e depois

começaram a cuidar do deslocamento, da Inspeção de Saúde Pública nos Portos. Especialmente no Rio de Janeiro que foi o local onde eles escolheram se estabelecer. E aí a evolução disso, após o fim da monarquia no Brasil que é 1989; a gente tem a Revolta da Vacina, ficou famosa, hoje voltou a uma grande discussão. Aqui a Revolta da Vacina, ela veio a dar um poder de polícia que é onde a gente vai levar essa discussão: dar poder de polícia para as autoridades sanitárias, de imposição de captura de ratos no domicílio, até então não existia muito estabelecida essa lógica do homicídio inviolável... e vacinação em massa. O uso da força com o uso do poder repressor do Estado (que é a força policial mesmo né). No Brasil em 1985 a gente vê a criação da Vigilância Sanitária e Epidemiológica que amadureceu de alguma forma a área. O estabelecimento da Vigilância em Saúde Mais adiante só foi possível após a criação do SUS lá fora a rede informatizada de Vigilância Sanitária muito importante e da Vigilância Epidemiológica também.

• 34:29 - 35:04
A implantação do Sinan do Datasus tão logo a tecnologia de transferência de dados permitiu isso, e o aprimoramento constante foi acelerada e progressiva, e até por volta de 2000 2006 2007. O Estado de Minas Gerais, que é muito populoso, demorou um pouco mais a implantar uma rede que tivesse um nível de informatização interessante e o aperfeiçoamento dessa rede de vigilância em saúde que culmina na lógica da pandemia que nós vamos abordar mais adiante que nada mais é do que o monitoramento do indivíduo doente. É a identificação de preferência precoce (extremamente precoce dos contatos desse indivíduo). é um objeto de trabalho muito importante muito relevante para a vigilância epidemiológica. Então dito isso e eu falei de mais, como vocês estão convivenco comigo desde março e devem ter percebido, sou muito prolixo. Eu peço desculpas, e deixo se alguém alguém tiver alguma colocação a fazer. Nós vamos agora... Dito isso, se Apresentador 2 também quiser falar alguma coisa, na nossa programação nós vamos passar os vídeos. São quatro vídeos curtinhos, para vocês assistirem e depois a gente abre discussões. São relacionadas especificamente ao monitoramento moderno, é isso que a gente conhece hoje a partir do uso dos smartphones. Ok?

E3. Eu já trabalhei com dados de pacientes com doenças mais graves. O uso do SINAN, o informe par as centrais com o nome da pessoa, eu sempre entendi como seguro. Agora a observação da pessoa pelo celular eu tenho um grande pé atrás. Não precisa chegar a tanto.

E7. Já falei que eu não instalaria(...) continuo com a mesma opinião. Até que me provem que funciona cem por cento e que todo mundo instalando faz a diferença na pandemia, eu não mudo de ideia. Nem recomendopros meus pacientes.

• 36:05 - 36:42
Apresentador 2: Ok. considero que é muito importante traçar essa linha histórica porque a gente até brincou quando a gente estava conversando. A gente não é dessa era da tecnologia. Da era da tecnologia são os nossos filhos: estão aprendendo primeiro a digitar antes de escrever. A gente usa o computador, mas continua escrevendo a nossa história, ela está sendo construída ao longo do desenvolvimento de tudo isso. E a gente resgata a história de como nós chegamos à capacidade do rastreamento hoje a gente considerou importante. Espero que para vocês aí também tem sido bom proveito e a

gente está à disposição aí para um questionamento para a gente passar para a próxima fase.

E2: Vou colocar um questionamento aí do Apresentador 2, uma questão para vocês e para os demais na seguinte perspectiva: o que motiva tudo isso? Qual é a motivação? Isto é algo planejado? Isto é algo aleatório, ou algo que aparentemente é aleatório sem uma motivação específica, mas ele é da essência do ser humano? O que motiva?

Apresentador 2: Vamos aguardar os colegas...

Não, quem vai responder são eles... Vocês estão altamente preparados.

E5: O que motiva esse controle das movimentações?

E2: É que o Apresentador 1 e a Apresentador 2 fizeram uma exposição de como era antigamente como é hoje. Então assim nós saímos de, teoricamente, uma era ultrapassada e estamos na era legal né. Isso foi planejado? A gente poderia estar em outro local gente poderia estar mais "avançado" do que estamos hoje em dia, ou ainda poderia estar na Idade Média?

E5: A julgar pela explanação que Apresentador 1 fez ao longo desse tempo aí a gente vê esses controles né. Esses controles migratórios na verdade né. Controle de passagem deslocamento. Eu vejo que tudo tem várias facetas. Você tem uma faceta da preservação mesmo, do ser humano. Por exemplo se eu tenho pessoas doentes, tem que controlar realmente, senão espalha para todos os lugares e chega numa bola de neve que você não consegue mais lidar: como ele até colocou ali: no período da hanseníase, se você tivesse um controle de cura e como a gente vai ver que nos dias de hoje às vezes os vírus vão trazer outras coisas, como a pandemia da covid. é uma tentativa de você preservar o ser humano mesmo. Mas também tem várias facetas da mesma questão. As questões econômicas,, as questões que se relacionam com isso. Eu não vou só pensar por exemplo quando eu limito por exemplo o deslocamento...

A gente está vendo por exemplo nos Estados Unidos, movimento de refugiados, .Alguns são refugiados mesmo outros são simplesmente migrações né não necessariamente refugiados de guerra. Se houvesse para os refugiados condições sociais e não inóspitas de outros países. Na verdade você tem também uma política de preservação do mercado dos recursos do país, econômico.. se chegam os imigrantes, aqueles imigrantes terão que trabalhar, utilizarão recursos econômicos... então tem muitas coisas que são envolivdas desses controles de deslocamentos.

Temos aspectos da saúde mas tem também aspectos sociais econômicos e de recursos do país dos países. é como se cada um tivesse defendendo o seu espaço. Eu vejo dessa maneira também.

E2: Sim E5, é tão assim. Não vou me render muito isso novamente não que ainda tem muita coisa pela frente mas eu só queria colocar isso aí. Não é aleatório. Mesmo que o planejamento não seja estratégico... apesar de vários momentos ter planejamento

estratégico no meio desta pseudo aleatoriedade. Mas poderia sintetizar a sua fala num dilema que estamos vivendo hoje, entendendo duas ou três grandes áreas: da saúde que não é exclusivamente biológica, ela também é social e ela também é ciências humanas. Então, na saúde ela também é tecnológica. A saúde é uma outra área que também não é exclusiva que é a da economia... que também é social que também é humana. O dilema que hoje nós temos é a economia de saúde. Vamos quebrar o país para garantir a saúde,ou vamos ter saúde depois de conquistar a economia, hein?

Apresentador 1: Alguém tem mais alguma consideração pessoal senão não passam um primeiro vídeo tentar fazer que a projeção de. Ao minuto. Primeiro vídeo pessoal ele trata de uma campanha e explicando como funciona o rastreamento de contatos. É uma campanha da ONG gente achou um vídeo muito didático importa que vocês tenham acesso até para a gente continuar as nossas discussões.

Ele é bem curtinho. Vou projetar pra nós aqui.

EXIBIÇÃO PRIMEIRO VÍDEO

• 45:02 - 45:19
Agora é que vou projetar aqui. O nosso segundo vídeo. E fala sobre o impacto da pandemia. A questão foi até suscitada pelo E5 quando falamos de refugiados e imigrantes.

EXIBIÇÃO SEGUNDO VÍDEO

• 50:03 - 50:07
O nosso próximo vídeo pessoal.

EXIBIÇÃO DO TERCEIRO VÍDEO

• 50:14 - 50:30
E esse é um vídeo que mostra como funciona a monitorização de contato com pessoas por dispositivos. Pelo iPhone. A gente gostaria que vocês se assistissem também como ocorreu no jogo aqui.

EXIBIÇÃO DO TERCEIRO VÍDEO

• 1:00:31 - 1:01:07
Então pessoal a gente fez uma apresentação desses desses três vídeos que a gente achava que era importante. Fazer uma revisão de conceitos deles né. Mais adiante depois que a gente começar essas discussões vamos falar um pouquinho sobre o que dizia a legislação brasileira antes e quanto foi mudado até agora. Pouco tempo antes da

pandemia a gente teve duas legislações que são tidas como um marco para esse tipo de monitoramento. Mas primeiro a gente queria saber as impressões dos colegas a respeito de questões que são muito antigas para Bioética.

• 1:01:08 - 1:01:23
Primeiro a questão do sigilo. Vocês acham que o sigilo dele é preservado no uso de tecnologias de rastreamento? Em que medida? A palavra é franca para vocês, podem ficar à vontade para falar.

• 0:00 - 0:00

• 1:01:37 - 1:02:11
E5: Olá gente, eu vou falar. Eu acabei instalar o programa para testar, tá falando aqui que não tive contato com nenhum nem me exposto, mas eu acho que ainda é um recurso frágil né. Por conta talvez da divulgação, da acessibilidade ao aplicativo, do recurso disponível.

• 1:02:12 - 1:02:44
Eu acho por exemplo que eu tive contato,totalmente paramentada, então em função de meu trabalho. Eu vejo, eu acho... que o sigilo desde que você tenha nenhum objetivo claro pelo qual ele esteja sendo feito. Por exemplo: você tem um risco de danos a terceiros. Como a gente vê no caso da pandemia, vamos pegar a pandemia em si.

• 1:02:45 - 1:03:19
Que você tenha a preservação de dados pacientes, apenas para pessoas que estejam ali. Quando a gente manda nossas fichas de notificação para a vigilância sanitária estou de certa forma quebrando o sigilo da consulta mandam lá para vigilância para uma pessoa que não estava naquela consulta. Porém estou mandando também para um órgão que também tem a sua obrigação de proteger dados do paciente.

Então você de certa forma se informa que também tem suas suas obrigações éticas no trato com os dados. Eu não vejo isso como uma quebra, entendeu... Igual o uso do aplicativo, ou o “não uso” do aplicativo. A gente vê países que estavam utilizando esses recursos também. Já vi outras notícias, utilizar assim, de uma forma bem maciça mesmo para fazer esse rastreamento. Você tem uma proteção de dados individuais. Por exemplo passei para lá que Apresentador 1 tinha um teste positivo e sei que foi Apresentador 1 porquê eu tive uma informação que me chega.. pra mim, mas preservando o dado. O que eu sinto como uma possível fragilidade. Talvez a gente tenha fragilidades do sistema. Por exemplo a gente viu há um tempo atrás aí outra quebra de hackers que entraram e vazaram dados pessoais de milhões de pessoas, dados bancários.

Então aí essas essas infrações, esses crimes cibernéticos que podem ser uma fragilidade. Mas eu acho que você fazer essas notificações para outros essa é uma coisa importante. Se a gente não consegue controlar, por serem doenças de notificação compulsória.

• 1:05:05 - 1:05:11
APRESENTADOR 2: Alguém mais deseja contribuir... Excelente contribuição.

Vamos passar pelo próximo então, Apresentador 1?

Apresentador 1: Isso se ninguém tiver mais nenhuma consideração a fazer. A gente colocou um paralelo da questão. A gente começa falando sobre sigilo da bioética a gente pensa numa proximidade muito grande entre sigilo e anonimato, e permeando isso a tal da confidencialidade. A confidencialidade está muito relacionada à relação pessoal pessoa médico, paciente e equipe de enfermagem ali próximo.

É algo que a pessoa diz de si, é algo que ela diz de si, do seu processo mórbido... Na maioria das vezes é que não tem implicação legal alguma desde que não envolva prejuízo a terceiros nem prejuízo a outra pessoa. Comprometimento da integridade física moral ou social de outra pessoa que justifica essa quebra de confidencialidade. Essa discussão ela começa a ficar, a borbulhar no Brasil a partir da pandemia do HIV.

• 1:06:33 - 1:07:09
Havia uma necessidade muito grande de proteção, à qual outras doenças estigmatizantes, as chamadas doenças negligenciadas, elas não tiveram a chance de fazer isso, porque elas cairam no domínio público, no período onde o uso da tecnologia... ele não existe. Como por exemplo a hanseníase, o próprio transtorno mental que é muito difícil de se manter uma confidencialidade irrestrita de todos os tipos de transtorno mental, pq que implicam intervenções que são comunitárias.

• 1:07:09 - 1:07:45
Eu moro no interior trabalhei na saude mental, sei muito disso. A visita domiciliar do CAPS e é rastreada pela vizinhança; no paciente isso causa uma série de constrangimentos... e às vezes até a própria interrupção ainda que temporária do processo terapêutico de parte do paciente. A gente tem também é junto com essa questão de confidencialidade. Algumas literaturas trabalham a questão da espontaneidade de demanda: o indivíduo, ele participa se ele quiser.

• 1:07:45 - 1:08:23
Se ele estiver à vontade para aquilo, se ele se sentir seguro. Me parece essa é uma questão importante no sentido do aplicativo de rastreamento e na própria construção democrática. Na construção democrática, porque veja... num país democrático a gente... não consigo conceber a instalação do aplicativo nos smartphones como uma condição obrigatória. O indivíduo precisa ser convencido a isso. A moça (do vídeo apresentado) ela faz um trabalho aí nesse vídeo de nove minutos de conversa e a pessoa olha é muito bom e ela mostra apenas pontos positivos.

• 1:08:24 - 1:09:06
"É muito legal porque você vai instalar o seu celular, e você vai fazer". Existe um parâmetro ela apresenta apenas pontos positivos da proposta e eu acho que eu percebi que nesse vídeo, até conversei sobre isso com Apresentador 2.. Me parece que é um processo que vem depois das ações que foram tomadas em vigilância epidemiológica oriundas da da pandemia do HIV. E a gente começa a ter uma relação de discutir amplamente no serviço de saúde questões de confidencialidade, anonimato, sigilo, demandas espontâneas estruturais, de uma forma onde não existia no mundo e uma

tecnologia como essa que nós temos hoje.

• 1:09:07 - 1:09:48
Né. E eu me lembro de trabalhar no CTA a quando eu era estagiário em 2000 e 2004. Nós fazíamos os atendimentos das pessoas que iam para fazer testagem utilizando um registro que era numérico. Era no papel, mas era numérico e as iniciais do nome de alguém então cruzava um número, com as iniciais do nome para a idade da pessoa. Aí ,para entregar o resultado, a gente ficava sujeito a isso para que o indivíduo não fosse identificado, e que não fosse solicitado de forma alguma um documento de identidade ou CPF.

• 1:09:48 - 1:10:34
Era era uma tentativa de estimular a testagem do HIV como se colocasse na mesa o sigilo como atrativo. Você pode vir, entregar seu exame, receber o resultado, ninguém vai te pedir nada. Acho que até hoje a gente utiliza uma versão mais moderna do sistema, do SI -CTA. Faz um tempo eu não opero, mas o sistema utiliza essa lógica: que é um cruzamento de um número de série que é emitido para aquele indivíduo que testou, mais as iniciais do nome, mais a idade. Sem documentos, sem identificação e, o que eu acho mais temerário em alguma medida e sempre fui muito contrário a isso,.

Era um dos programas que o Governo Federal utilizou como o “Fique Sabendo” que era fazer a testagem em massa de pessoas em praça pública ou em feiras,, ou em parques, eventos públicos. Eu achava muito, muito complicado fazer isso sempre. Era resistente em aderir a esse programa, porque você não tem o tempo necessário para um pré atendimento, pré aconselhamento, pós aconselhamento.

• 1:11:07 - 1:11:50
Esse indivíduo é tomado um dia por um diagnóstico. Sei lá.. A pessoa viveu uma situação trágica de um falso positivo. A pessoa pode sair dali e fazer alguma coisa consigo mesmo, porque foi mal orientada ou porque ele tem uma percepção e errada do que é a testagem sorológica positivo para HIV. Pode ir lá e acabar com a própria vida. Quando trabalhei no CTA a gente teve um caso assim, de uma pessoa que foi atendida no serviço nesse ano por acaso; não era eu que estava, mas eu atendi semanas depois um rapaz que se dizia Irmão de alguém que teria feito um teste.

• 1:11:51 - 1:12:23
E ele estava lá porque ele queria ver o teste do irmão. Aí ele falou “não, meu irmão, ele esteve aqui, fez o teste.... Eu queria ver”. Eu falei pra ele: Olha se ele fez isso ou ele não fez, não sei quem é... Mas para ter acesso só com intervenção do juiz. Ele suicidou ontem. A gente queria saber se ele se suicidou porque ele descobriu que ele era portador do HIV.

• 1:12:25 - 1:12:59
Ele deveria judicializar isso. Não podia dar essa informação, mas a gente olhou nos registros. Na verdade ele tinha feito realmente a testagem, só que tinha dado negativo. Na época o método ainda era o ELISA, então tinha confiabilidade. Embora existisse janela imunológica considerável, então eu penso que essa lógica do rastreamento para

mim, e por tudo que a gente estudou, ela é um aperfeiçoamento disso.Talvez seja um aperfeiçoamento histórico do monitoramento de contato. Daquele de chagas, por exemplo. Descobrimos um caso de Chagas para fazer um bloqueio no quarteirão. Não sei se os colegas já trabalharam com saúde coletiva nessa medida,mas no interior ainda é muito comum em bairros muito afastados, bairros próximos de área e rural e mata que, se e encontra um caso positivo de achar que se faz um bloqueio na família e no bairro. O pessoal de Controle de Zoonoses vai sem ordem oficial, conversa com as pessoas pede autorização para entrar nas casas para tentar fazer captura do barbeiro passa orientações às pessoas no domicílio.

• 1:13:45 - 1:14:16
Então já é uma evolução disso e talvez se a gente possa puxar mais pra trás. Segundo Michel Foucault, Gosto muito desse livro.. “Microfísica do Poder” se não me engano, capítulo 6. O Michel Foucault era psicólogo e filho de médicos. E ele escreveu muito sobre genealogia da doença, do adoecer, de alguma forma... Está nesse livro no capítulo 6, “O Nascimento do hospital”.

• 1:14:16 - 1:14:52
Ele fala sobre isso que na lógica da saúde coletiva dos deslocamentos: antes o deslocamento era feito pelo médico. Ele ia até a pessoa de cavalo, consultava a família inteira, visitava uma comunidade... Depois com a compartimentalização do hospital, então a pessoa vai até o lugar do hospital e aí depois vem a saúde da família para reverter isso. Daí você tem uma equipe que não é necessariamente a figura do médico, mas a equipe vai até a casa da pessoa.

• 1:14:52 - 1:15:31
Esse exame de contato, antes ele era feito dentro de uma lógica domiciliar. Depois isso foi meio perdido em algum momento da história. E isso é retomado, primeiramente no Brasil a partir do da proposta do SUS. Então eu acho que... não sei se eu consigo ser claro, mas para mim são períodos históricos de evolução do mapeamento, na lógica da história natural das doenças, de identificar os indivíduos e fazer esse bloqueio por uma necessidade que aí fica critério de vocês pensar.

• 1:15:33 - 1:16:08
E2: Apresentador 1, nós temos aí alguns elementos que você mesmo trouxe e que a gente poderia discutir se há algo de novo nos elementos da bioética. Primeiro você começou a falar no anonimato, confidencialidade, espontaneidade e, vou falar o inverso: de espontaneidade de demanda. Então nós hoje como profissionais... você está lá no seu consultório, você está no hospital, ou em alguma policlínica (não se aplica para o setor da família).É até uma crítica com relação ao estabelecimento de relações: A pessoa precisa; Ela pode ir em A, B ou C. Ela foi evidentemente pela necessidade dela, mas ela foi espontaneamente. Ela poderia ter ido em B ou em C e D. Aí a crítica À saúde da família, no território seria atenção primária. Se ele gosta não gosta de quem está lá naquela equipe, ele não tem escolha. Ele tem que ir lá. Então aí a questão da espontaneidade fica prejudicada.

• 1:16:44 - 1:17:29

Mais uma vez a pessoa chegando no no serviço que ele teria possibilidade de ir em outro. Então você não foi lá e resgatou ele. Ele foi espontaneamente, mas ele foi espontaneamente em cima de pressupostos. Pelo menos três que você colocou ali. Primeiro que ele pode fazer os relatos, comentários, assegurando um certo anonimato para não expor ninguém e de forma que não atrapalhe o raciocínio clínico, o diagnóstico e tratamento, garantido a ele. Isto é também garantido a ele que aquelas informações, naquela relação quando estão sendo tratadas, estão assegurados pela confidencialidade.

• 1:17:30 - 1:18:03
Então ele foi espontâneo,, assegurado anonimato e que teria confidencialidade... teria confidencialidade e que tudo aquilo que for tratado pode... Aquilo terá que ser feito um registro, com outras finalidades. Primeiro, o médico, de acompanhamento ao longo do tempo... e também em termos de comunicação entre profissionais em termo de período de e até mesmo questão judicial. Também de registrar de fato o que aconteceu naquela contrato estabelecido, na medida em que um procurou o outro.

• 1:18:05 - 1:18:46
Só que, aquelas informações, aquele relacionamento, o que gerou ali... precisa ser mantido sigilo.O sigilo é de responsabilidade do profissional e da instituição. Eles têm que guardar aquilo de tal forma que ninguém possa ter acesso ou todos os que estão envolvidos também tem que ter a perspectiva... não mais da confidencialidade, porque eles não obtiveram da relação com o outro e sim por intermédio do trabalho, que aplica se ao técnico de enfermagem, enfermeiro, fisioterapeuta e a todos os outros profissionais e biomédicos que ali estão. O pessoal da limpeza, pessoal da segurança, todos eles, de certa forma, poderia ter acesso ao prontuário, seja eletrônico, seja físico mediante acesso por senha ou pelo espaço físico. Todos esses profissionais eles são responsáveis e a instituição nesse caso é a que tem que assegurar que estes outros profissionais não vão abusar preceitos profissionais que eles têm, e de forma que Não deixe de ser garantido até mesmo se houver uma ordem judicial, para que seja aberto alguma informação em última instância. O profissional pode alegar o sigilo profissional e não revelar isso, vai até no supremo de forma que o sigilo é primordial no estabelecimento da relação de trabalhos de forma que estas questões sempre existiram e recentemente, melhor não tão recente, bem da história é que com a incorporação da tecnologia e da possibilidade de você ter acesso fácil rápido e de muitas pessoas e de muitos dados e mais você colocar essas informações e algoritmos, deve traçar um perfil de profissional de pessoas de poucos canais de comportamento e conduta.

• 1:20:11 - 1:20:45
E mais isso poderá ser hackeado e disponibilizado para parceiros com outras finalidades comerciais e econômicas, criminais e qualquer que seja o problema que teoricamente sempre existiu. Ganhou amplitude e sem perspectiva. É aí que vem a questão da Lei Geral de proteção dos dados. A LGPDI. Mas essa é a Lei Geral de Proteção de Dados. Então assim na essência na essência...

• 1:20:46 - 1:21:27
O que a gente está trabalhando e discutindo são questões que vão colocar vocês e aparecer no vídeo. Creio que vocês discutem primeiro a liberdade. Nós temos que

assegurar a liberdade, às pessoas... e à individualidade. As pessoas têm que ter qualidade em relação às suas individualidades. Porém não pode prejudicar terceiros. Meios tem que ser encontrado que para o indivíduo também não seja discriminado e que não sofra. Esta individualização então não é simplesmente pelo fato de eu não podem prejudicar terceiros que eu vou identificar individualizar discriminar ou pior. Recriminar, esta pessoa passa a ser estigmatizada. Então não é porque a pessoa é um traficante que durante a assistência à saúde ele será repreendido, ele será estigmatizando. Isto aí é não pode, deixando no mesmo campo de execução:

• 1:22:11 - 1:22:42
Não é porque é mulher, porque é negro, porque é pobre, porque tem alguma deficiência física. Nós vamos discriminar esta pessoa e não vamos assegurar ele, a essa pessoa, os direitos fundamentais que todos os outros: homem branco, hetero,, rico, trabalhador,, em produção, jovem, tem em relação aos demais... Criança, mulher, negro, deficiente e idoso.

• 1:22:43 - 1:23:21
Então a questão ela está muito mais relacionada à essência do ser humano no relacionamento com as outras pessoas e com o meio ambiente. Porque a tecnologia disponível hoje ela se assemelha a o que foi no passado, ao que foi o fogo,, a invenção,o da roda ou mais recentemente, uma faca. A gente tem pode decidir se a faca serve para descascar uma laranja ou pode usar a mesma faca para matar uma pessoa. Então nós a bioética trabalhamos com a essência do ser humano e o ser humano tem fundamentos que... eu coloquei no início qual a motivação da gente sair de onde estavavamos para chegar até hoje. Mas os fundamentos históricos são os fundamentos de hoje. Então. A pergunta que eu deixo para vocês: Será que a essência da mente do homem de hoje é diferente do homem de alguns séculos do passado? ou do homem na América Latina, da América do Norte, da Europa,, da ásia e da áfrica? são diferentes? Na verdade eu estou citando uma pessoa, mas é a turma.

• 1:24:16 - 1:25:06
Apresentador 2: Então professor eu achei excelente essa colocação tem até algumas anotações que a gente fez para poder passar aos colegas e trazer assim até para próximo da gente. A gente tentou resgatar a de la pra atrás e trazer para a nossa realidade vivenciada para a gente até fazer esse paralelo. Essa importância da bioética nos processos, independente de nós estarmos lidando com a tecnologia ou não, porque o que a gente entende é que a tecnologia ela amplifica muito o que a gente vive hoje, mas a gente sempre esteve em processo de monitoramento e rastreamento do que vem ocorrendo na evolução história humana. Por exemplo: nós temos hoje para o monitoramento dos casos de covid um sistema nacional. Ele se chama GAL: Gerenciador de Ambiente ambulatorial onde todos os exames que são feitos independente pelo serviço público ou particular eles são levados para esse sistema, dando às autoridades a informação de que ali tem um indivíduo infectado e os dados daquele indivíduo.

• 1:25:40 - 1:26:26
Eu tive por exemplo uma experiência onde eu suspeitei de estar com o vírus... fui até

um serviço particular. Fiz o exame, e passados cinco dias eu fui contactada pela Secretaria Municipal para falar. Nós tivemos a informação aqui que você fez o exame, que seu diagnóstico foi positivo. Então às vezes é feito de uma forma que não é tão visual como ter um aplicativo no seu celular ali na palma da sua mão, mas isso está acontecendo naturalmente dentro do processo.

• 1:26:26 - 1:26:57
Então dentro da lei do sigilo, do cuidado com a utilização dos dados na internet... O Marco Civil da Internet prevê a individualidade. a restrição ao acesso às suas informações pessoais, mas também prevê que quando se trata de algo que impacta no todo e que, por exemplo, um problema de saúde pública onde podemos ter toda uma sociedade comprometida.

• 1:26:57 - 1:27:30
Então libera- se essas situações de gerenciamento de dados como por exemplo a gente está acabando de vivenciar que é o controle de onde nós temos o indivíduo infectado. E aí quando a gente tiver um aplicativo como esse onde eu pessoalmente tenho que ir lá baixar e informar se eu tenho o vírus se eu estou com ele ou não, ele tem ele caso a informação com sistema nacional porque eu poderia estar mentindo.

• 1:27:30 - 1:28:06
Então como é que a contraprova vai caso essa informação com um sistema nacional se real ou não. Sim após a ativação do meu exame. Então o que eu vejo é que a internet ela da amplitude do processo que a gente vivencia mais que de uma forma ou de outra. Nós estamos o tempo inteiro monitorando as situações e rastreando por um bem maior, que seria o controle da doença favorecendo a preservação humana.

• 1:28:06 - 1:28:43
De uma forma ampla. E nós estamos trazendo agora por exemplo a situação para o COVID, mas nós tivemos aqui há poucos anos atrás,, seis anos o zika vírus que foi um boom que aconteceu, que não se esperava, a chicungunha veio junto. E aí enquanto nós tínhamos uma gestante que estava com o problema a gente monitorava toda uma região para que nós tivéssemos um mínimo de controle da endemia naquele território.

• 1:28:43 - 1:29:18
Então são situações que a gente vive em que a gente vive hoje com que eu percebesse que a internet é uma bem pra poder ampliar os cuidados que se tem que a história gerou que aquela primeira colocação que você fez aqui. Será que ela é aleatória? Aleatória ela não é porque a gente não preserva a humanidade...Mas é planejada ou adaptada? Então eu acho que a gente vem adaptando esses processos aí ao longo dos anos.

• 1:29:20 - 1:29:22
Mais algum colega que quiser contribuir....

• 0:00 - 0:00

• 1:29:29 - 1:30:12
E5: Isso eu vejo assim a tecnologia na realidade às vezes a tecnologia, ela é muito demonizada... na verdade a gente tem que usar a tecnologia de forma a melhorar,, por

exemplo a agilidade que a gente pode ter nas notificações. Os problemas de notificação. Não estou falando só COVID 19, mas a gente tem nas notificações, e gerar subnotificação, porque as fichas de notificação são grandes, são demoradas, e o médico ou enfermeiro....

• 1:30:12 - 1:30:45
Quem tiver prestando assistência tem que preencher... a questão que é papel enorme de frente e verso. Então vezes aquilo ali ele acaba ficando meio que com preguiça ou com pouco tempo, não faz. Então isso é um problema dentro do serviço de saúde. A tecnologia poderia fazer isso ser mais rápido. Na forma de cliques ali a notificação já vai direto para a secretaria. A gente tem as casas que tem que ser feita 24 horas.

• 1:30:45 - 1:31:34
Um exemplo aí com o vídeo de 24 de junho já tem que ter mandado os das secretarias ganhando sarampo também tem que ser dentro ali num prazo bem curto. Então eu vejo que tecnologias podem ser um recurso muito importante para a gente né. Agora tem a questão da esses aplicativos de celular como foi mostrado aí. Ele envolve aquilo que o professor falou, que é da espontaneidade. Depende da pessoa querer usar. que ele recusou e o Apresentador 1 tinha comentado sobre isso. é que eu percebo para algumas coisas a vigilância funciona muito bem. A minha mãe....

• 1:31:34 - 1:32:08
Esse ano ela teve um acidente com um escorpião, e daí vieram e fizeram notificação. Três dias depois o pessoal vigilância veio em casa para saber o que tinha acontecido, como que tinha sido acidente,, onde tinha sido então assim todo o trabalho é fundamental. Acho que faltou por exemplo dentro da.. Aí eu não estou culpando necessariamente a vigilância, mas eu acho que faltou por exemplo dentro da contenção da COVID

• 1:32:09 - 1:32:55
A gente ter até mais RASTREAMENTO MESMO. A gente ter uma forma de fazer o bloqueio igual Apresentador 1 falou o que fazer quando se tem os casos de Chagas, talvez até pela velocidade que a doença tem de expansão. Mas a gente viu outros países em que isso foi feito de uma forma mais maciça mesmo. A gente tem um exemplo que foi lá na Coreia do Sul estado de estatizar. Pegava o pessoal que tinha tido e colocava lá uma quarentena no local da quarentena lá dentro de casa todo mundo conhece todo mundo. Aqui não, tá com COVID, sai pra rua, não olha você nem atende os pacientes lá, se não tem lá que ele vai fazer isolamento.

• 1:32:56 - 1:33:30
Quem vai olhar se está fazendo isolamento mesmo? Então acho que talvez tenha faltado a vigilância em relação ao cumprimento dos autoisolamentos,, porque aqui o que se vê no Brasil e em várias partes do mundo foi por altas ordens ou seja depende do quê da espontaneidade, depende da vontade da pessoa também. Mas nós temos que pensar: será que isso vai ferir o direito de ir e vir? no momento que eu falo: Olha, tem que ficar em casa mesmo, que está contaminado. Será que isso aí vai estigmatizar aquela pessoa? Mas o bem da coletividade... será que mais mortes não teriam sido evitadas?
• 1:33:44 - 1:34:26

E2: Entrevistado 5, é verdade. O que você está dizendo agora. Depois que a gente começou a trabalhar com pesquisa qualitativa pela própria atividade profissional como médico homeopata a gente valoriza muito a fala, algo com muito significado e é uma análise da cultura em cima da hermenêutica. Tem uma questão que é crucial ,e a gente estava no livro de Mateus: Ninguém serve a dois senhores; ou você vai agradar um e deixa o outro chateado ou vice versa. Isso pensado de forma universal, abrangente, é complicadíssimo.

• 1:34:29 - 1:35:01
E aí na sua fala. Desde aquele dia que falamos, e você fala com frequência, mas não é porque na verdade era assim a questão do negócio. Porque de fato é um negócio! é um negócio e a gente o tempo inteiro. Tem que negociar o tempo inteiro, o tempo inteiro. Tem que negociar. E na negociação muitos são os fatores que estão envolvidos. Mas o problema não é o negócio.

• 1:35:01 - 1:35:33
O problema não é a negociação. O problema é o desejo de levar vantagem. O problema é a cobiça. O problema é de falta de domínio próprio, porque é praticamente impossível a gente relacionar sem negociar, sem negócios. Então não é a tecnologia que é o problema e muito menos o negócio que é o problema.

A questão somos nós e aí a questão é o quanto que nós. O que nós estamos disposto a doar. Para que a gente tenha uma vida mais Sadia SA Dave é melhor para todos aí que a gente tem que pensar nas periferias. Cada questão da conceitual bioética intervencionista e o quanto que nós teremos que pensar nos excluídos o quanto nós temos que pensar no excesso que nós temos e nos estragos que nós fazemos em detrimento dos óculos as viúvas dos estrangeiros dos pobres e necessitados.

• 1:36:19 - 1:36:50
E o quanto que a gente aí vem toda uma filosofia toda uma história toda uma administração uma ciência em relação a quem é pobre porque é pobre que é preguiçoso porque não trabalha e o Rico ele é rico porque ele trabalhou porque ele se esforçou tem mérito. Mas não é verdade. O que tem é a questão das oportunidades. Tem a questão da corrupção e tem a questão da cobiça e da opressão popular.

• 1:36:51 - 1:37:24
Se nós formos militar vamos dessa forma e na perspectiva de proteger de resguardar. Mas também sem também que o Brasil é do outro. Você fica na perspectiva que a gente vê muitas vezes a criação de dependência e de ações paternalistas e que você não desenvolve. Então a questão nossa da bioética é no nosso dia a dia e moderação. Nós temos que buscar o equilíbrio e moderação.

• 1:37:24 - 1:38:00
Sabe então assim quanto mais a gente avança na perspectiva do desenvolvimento das capacidades humanas e tecnológicas mais acirradas e mais distantes fica os opostos. é mais difícil fica a possibilidade de alguém que sai de onde está e chegar no outro ponto, entende. Então a nossa discussão na perspectiva da bioética nós não podemos tirar ou esquecer dessas questões e é isso que está.

• 1:38:00 - 1:38:12
Basicamente isso será medida para a gente medi praticamente toda a nossa discussão em qualquer assunto em qualquer tema. Aqui a gente puxa para Bioética.

• 1:38:14 - 1:38:47

E5 :Isso me fez refletir sobre situações que eu vivi aí atendendo pacientes que foram suspeitos ali para a covid. E aí me fez criar aqui um colocar alguns pacientes a Nina analisando as falas a questão da oralidade né. E você falou tem de paciente que tinha tido a suspeita e era um paciente que era um servidor público com a estabilidade.

• 1:38:48 - 1:39:23
Propus a ele que precisávamos fazer o período de isolamento na época, para evitar o contágio de outras pessoas. O paciente aceitou muito bem tranquilamente. Outro paciente que eu atendi ele era funcionário de uma empresa particular e ele teve a fala. Ele falou assim mencionou dois atestada vou acabar sendo despedido e teve outro paciente que eu atendi que era um paciente autônomo, trabalhava não sei exatamente com o quê, mas era autônomo e falou mais como é que eu vou fazer? Envolve economia, e também o negócio saúde.

• 1:39:23 - 1:40:08
E7. Eu tenho que trabalhar, eu preciso comer. Isso é a demanda urgente, global. Temos que olhar para os necessitados, sem dúvida. Então mudou, você perceber essas diferenças aí, é uma mesma medida sendo colocada para três pessoas em protestos diferentes todos com a necessidade mesmo manter isolamento para preservar uma coletividade. Mas num contexto diferente então eu tive que usar no recurso além de trazer nossas responsabilidades também dentro da saúde pública para todos eles mas assim foram três situações que acabou que essa fala me fez refletir sobre isso.

• 0:00 - 0:00

Apresentador 1
• 1:40:15 - 1:41:01
E4. Tenho uma outra consideração pessoal que tinha pensado eu gostaria de ouvir aí os colegas sobre qual seria o tempo de manutenção desses dados de rastreamento uma vez que há uma proposta de monitorização pessoal e a gente vê na dinâmica que a dinâmica funciona mais ou menos assim. E os dados deles são triangulares, essa triangulação é feita a partir do princípio que você tem três torres que vai conseguir que vão conseguir marcar o posicionamento do indivíduo com a precisão maior do que necessariamente o GPS.

• 1:41:03 - 1:41:38
a polícia utiliza muito esse essa ferramenta para identificar um indivíduo local de acesso celular com a precisão maior do que é o via satélite. A tecnologia de triangulação ela no Brasil não tem um uso amplo assim municípios pequenos por exemplo tem uma rede de celular e uma antena se precisar precisaria ter no mínimo 3 para poder fazer um monitoramento mais preciso do indivíduo. E quanto tempo por quanto tempo esses dados ficariam aí ainda que criptografados.

• 1:41:39 - 1:42:13
Isso vai entrar diretamente na discussão da legislação que o **E8** mencionou da Lei Geral

de Proteção de Dados e que essa é uma discussão que ela não fica muito estabelecida porque depende do tipo de tecnologia depende do tipo de decodificação que é feita. Então eu queria ouvir a opinião dos colegas porque a gente tem uma dimensão e aí isso fica para pensar. A gente tem dimensão coletiva na saúde no entendimento de que é esse tipo de uso de tecnologia vai favorecer o coletivo, a pessoa pessoalmente também a ter informações a respeito de onde ela entrou em contato com alguém contaminado. Hoje não. Mas esse tipo de rastreamento ele também é invasivo a vida particular das pessoas sob o ponto de vista da psicologia. Isso fere o eu oculto. Do indivíduo. E o marido que saiu falou com a esposa que ia no supermercado mas ele passou no boteco tomou a cerveja e uma vez que ele foi rastreado isso vai ficar muito tempo lá registrado que ele tomou essa cerveja que a esposa ou alguém ou quem quer que seja será o neto dele vai ter ciência disso daqui a cem anos 20 30 anos. Então a gente entra num âmbito muito particular dou rastreamento. Que é a lógica do individual em pessoas que hoje em dia optam por não fazer uso de nenhum tipo de tecnologia porque eu não quero, não quero que ninguém saiba onde estou.

• 1:43:21 - 1:43:58
Às vezes a pessoa fala sim eu vou para um retiro final de semanas ou eu vou para um passeio e vou desligar o celular. Porquê. Porque eu não quero. Eu não quero ser localizado. Eu quero abrir mão do mundo que se apresenta para a gente a partir do dispositivo móvel. é uma escolha que é muito particular e que as pessoas às vezes fazem isso permanentemente às vezes fazem periodicamente para transporte como a Apresentador 2 falou, porque nós, nossa geração não nasceu desse contato com a tecnologia.

• 1:43:58 - 1:44:37
Que Eu Queria propõe essa reflexão para vocês. Esses dados armazenados eles devem ser utilizados como a tecnologia diz que eles são o mapeamento feito em bloco então o que cada indivíduo usou no aplicativo ele é nada mais que um pontinho de luz ali. E aí você conseguiria fazer um mapeamento utilizado com sucesso lá nos Estados Unidos está sendo utilizado como determinação de onde se vacina prioritariamente, onde nós vamos colocar os pontos com maior número de pessoas infectadas, então é aqui que a gente vai botar os postos para funcionar são essas áreas que são mais críticas.

• 1:44:37 - 1:45:07
E6 -Essas áreas de deslocamentos pessoas , acabou evidenciando que as pessoas nas áreas mais pobres eram quem mais deslocavam de um ponto a outro ponto por força de necessidade de trabalhar e tudo estariam ali também mais suscetíveis mais expostas à covid. O vídeo então a pergunta que fica é essa: Até quando esses dados devem ser guardados? E se de repente eles venham a expor apenas as pessoas mais pobres, as pessoas mais necessitadas? Tem que pensar isso daí.
Acho que mesmo em casos de emergência em Saúde pública devemos manter o sigilo e anonimato para cumprir os preceitos éticos relacionados aos seres humanos, né?

Apresentador 2
• 1:45:08 - 1:45:39
Quero colocar mais uma informação aí também, que a gente vê a produtividade da utilização de serviço muito em países mais desenvolvidos porque eles estão de disponibilidade da tecnologia como o Apresentador 1 citou aqui né. Eu preciso e antenas ou satélites interligados isso vai dependem da evolução de cada país.

• 1:45:39 - 1:46:22
Qual é a metodologia que ele utiliza para eu conseguir fechar esse circuito e essas informações elas conseguirem entrar na rede. E é aí a gente estudou aqui viu que nos países onde eles conseguem ter mais acesso a essa condição de tecnologia houve um impacto de até 30 por cento na redução da disseminação do vírus. é uma situação agora que nós temos uma outra situação que no Brasil a gente não consegue ter a amplitude dessa rede em triangulação todo o território brasileiro e nós temos um a outra.

• 1:46:23 - 1:47:01
Uma outra posição que foi tomada pelo nosso governo da descentralização das autoridades e decisões no âmbito da saúde é que ela pode ser estadual. Ela pode ser regional ela pode ser municipal. Então isso também seria dificultado para isso acontecer o massa porque esse é o meu território e se o meu estado não adere a essa política. Se eu passar de um estado para outra vou ter essa esse tipo de atividade comprometida então ela não vai funcionar naquele território.

• 1:47:02 - 1:47:40
Então dada a essa descentralização da decisão também é um pacto que nós teríamos aí para a nossa realidade. E tanto que quando a gente faz pesquisas a gente vê que quem está utilizando o serviço de monitoramento móbile é uma quantidade muito inferior à nossa capacidade da nossa população de uma forma geral. O último estudo que eu tinha eu consegui levantar que era de 2 milhões e 800 mil pessoas cadastradas.

• 1:47:40 - 1:48:16
Até tentei achar aqui mais outros estudos para ver se esse número já se atualizou. Mas você vê que é algo assim irrisório ao dimensionamento de um país tão populosos como é o nosso. Então queria colocar mais essa situação junto ao questionamento que o Apresentador 1 aplicou aqui pra gente pra embasar mas também na resposta e na contribuição de vocês. Nós não temos uma lei ou uma diretriz única no nosso território.

• 1:48:17 - 1:48:50
Isso também é dificultado para que a gente consiga disseminar esse tipo de prática de política pública. Isso se deu logo no início da pandemia. Então ela prevalece até hoje com seus estados tendo autonomia para as suas decisões. Estado ou município regionalização e nós temos muita regionalização do Serviço de Saúde.

• 0:00 - 0:00

Apresentador 1
• 1:49:00 - 1:49:22
E é aí pessoal baseado nisso que é que Apresentador 2 falou para preservação dos dados deve ser mantida deve ser mantida até quando, deve de eliminação instantânea. A criptografia de ponta a ponta que é hoje o método mais utilizada era segura para armazenamento de dados mapeamento o que vocês acham?

• 1:51:08 - 1:51:34
E6. Eu tenho uma resposta e não sei se e isso, se a gente guardar alguns dados relacionados à questão do uso do prontuário só para a questão de saúde pública eu não

tenho uma resposta mas acho até 20 anos seria em média.

E8

• 1:51:49 - 1:52:30

Obrigado enquanto você falava tentava fazer anotações e eu me recordei de uma situação que não era nem da era tecnológica ainda mais assim só exemplificando eu lembro que me incomodou um pouco. Certa vez eu estava passando numa dessas lojas que tirou xerox e aí tinha uma pessoa com um atestado médico de um paciente e com uma foto chocante ali pedi ajuda caso pessoas que passam em clínicas eu pedi ajuda para as pessoas doentes e aí há um atestado médico com data.

• 1:52:33 - 1:53:30

Observei estava um pouco rasurada e uma foto e quando eu peguei eu vi a foto de uma criança virei em cima do balcão de curiosidade e vi que era de 1981 e aí eu fico pensando só com relação aos e a pessoa parava de chorar na montagem ali na para poder pedir ajuda a fazer uma época que não era ainda dada a tecnologia como nós temos hoje, e isso foi há uns 10 anos que eu vi , uns 5 a 10 anos não lembro exatamente. mas é uma situação que eu me pergunto às vezes com esse uso das informações também é um ponto e outro ponto também na sua fala nessa discussão que nós observamos até hoje nós instalamos às vezes falam disso mais de um consenso geral diversos aplicativos e diversos aplicativos e quando você chega num determinado ponto você chega num determinado local e já faz o seu check ali relacionado diretamente à saúde. E aí nós entramos em discussão. Como monitorar isso. Agora já estamos de certa forma já utilizando aqui alguns, alguns até por não saber utilizar não irá em configurações como passou no vídeo como faz a atualização de configurar disponibilizam não apenas por ter instalado e quer utilizar o aplicativo WhatsApp ou aplicando o sim em todo o cabo disponibilizando essas informações e aí é outra questão que é quando é bem interessante porque eu faço essa nota em que uma nação de como eu utilizo as redes sociais, também há um tempo atrás digamos que não é exatamente o que está passando hoje mas é um exemplo de termos.

• 1:54:24 - 1:54:58

Até quando essas informações ficarão disponíveis e aí se você utiliza por exemplo a nuvem não há nada o drive do Google seja para registro de fotos ou colocá la para uma não ter perda de fotos ou configurar o celular para poder já salvar automaticamente passa se dez anos não um lembrete o vídeo seleciona os melhores momentos que você viveu durante ali 5 10 anos para receber um ou outro dia com as fotos com coisas que eu não me recordava que eu tinha tiradas as fotos que eu tinha com ela configurado para que ficasse a salvo da tela.

• 1:54:59 - 1:55:32

Então peço que corra junto com essa discussão pertinente um pouco nesse sentido mas aí trazendo para a questão da hora a gente percebe que tem informações da dados que de certa forma ela é importante para poder compreender o todo o contexto também né. Quando o professor traz aí até um exemplo é homeopata, eu fico lembrando eu gosto do tratamento.

• 1:55:32 - 1:56:27

E aí meus pais fazem o mesmo método que nos até 12 anos então existe também um histórico nos dados digamos que sistémico de compreender ali todo o contexto da da

Saúde Familiar do grupo familiar daquilo que nós temos ali também de onde é hereditário ou de questões próprias mesmo vamos dizer se ocultas no sistema de casas a gente nem nem lembra que aqueles aqueles problemas foram registrados e não penso que a personalidade nessa questão ela é bem pra discutir sobre ela tem um até uma questão assim bem relativa dos casos na questão eu acho que envolve mais talvez aí a proteção desses dados não onde esses dados vai ficar é a quem vai ficar disponível e quem vai ter acesso a esses dados.

• 1:56:28 - 1:57:09
Vai entrar aí como algumas publicações ainda num determinado momento virar domínio público. Eu acho que a questão é mais pontual nisso nenhuma proteção desses dados é arquivo externo. Durante um período até porque é sempre possível pensar por exemplo nós falamos aqui alguém falou em 10 a 20 anos mas imagine há 20 anos você quer meu próprio exemplo há 20 há mais de 20 anos eu faço um acompanhamento por um pedido de tempo ou deixei de acompanhar parei de acompanhar com esse mesmo médico.

• 1:57:10 - 1:57:42
E1. Dez anos depois ele volta retornar aquela mesma consulta e o médico tem o meu histórico daquilo que ele me tratou há 20 há vinte anos atrás. A idade era e não tínhamos sistemas as tecnologias que nós temos hoje então espero viver para pelo menos mais uns 40. Então possivelmente seria pra mim interessante esquecer que esse médico ele teria ali todos esses dados ao longo dos 40. Ao longo desses próximos 40 anos ou pelo menos isso estar.Quero que tenhamos a nossa privacidade preservada também. Isso pode descambar. E o governo tem que determinar as regras. Além do mais, quero ter o direito de ficar anônima.

• 1:57:42 - 1:57:51
Se tiver mais. Então esses são situações que acho que são muito específicas. Nesse sentido o que eu queria dar minha contribuição.

E2
Só pegando a questão que a **E8** falou em relação ao debate que vai guardar os dados tem uma questão que não foi abordada aqui é a punição, Para quem quebrar o sigilo . No meu ponto de vista semelhante ao da **E8** não tem tempo é atemporal. Aguardo os dados sobretudo hoje onde é possível guardar os dados praticamente em nuvens.

• 1:58:36 - 1:59:12
Antigamente não, na área de ciências contábeis que tem aquele tanto de papel, nem de guardar aqueles papéis de tantos arquivos gigantes, não se tinha tanto espaço físico. Então aí a questão da idade era basicamente o aspecto físico para guardar quer dizer que parte daquele material tem sentido um espaço gigante guardando 50 anos atrás. Mas hoje você não ocupa espaço praticamente nenhum. Então eu penso que não tem tempo. Porém quem não utilizar adequadamente os dados e usar indevidamente sem a permissão, essas pessoas precisam ser responsabilizadas e aí a questão está na responsabilização do uso indevido de dados. Meu ponto de vista, não sei se está no artigo, também não conheço a legislação completa e praticamente na perspectiva de visão geral do assunto para dizer que parte dos demais membros da sala, os alunos, as pessoas que estão aí na faculdade pode se manifestar conforme o pensamento deles, não

precisa ter medo de esta errando e nem ter vergonha um do outro aqui não.

Apresentador 1

• 1:59:49 - 2:00:24
É isso aí professor, seu raciocínio está em consonância com o que a gente tinha abordado porque tem uma relação muito próxima com a legislação. Eu acho que agora a gente já pode até falar sobre o Marco Civil da Internet. Como eu disse essas duas legislações foram semana passada, uma de 2014 e a outra foi mais elaborada de um marco em 2018 e uma reformulação recente proposta pelo Governo.
O Marco Civil da Internet era ele que era a chamada Lei Eduardo Azeredo.

Apresentador 2

Enquanto Apresentador 1 projeta, o Marco Civil da Internet é como se fosse realmente a base, da lei, é bem recente, de 2018 e esse Marco Civil ele foi discutido em 2014, então são discussões que a gente acredita que está em plena evolução e a gente tem visto mais do que nunca a necessidade dessa discussão e da evolução, tendo em vista o mundo globalizado e pluralizado que a gente vive, e esse marco que estabelece realmente não os princípios da garantia de direitos e deveres enquanto cidadão, enquanto seremos responsáveis pelo seu mundo, pela sua individualidade, mas sob o ponto de vista também de você relacionando com o próximo e com o mundo, como um todo, porque é o que a bioética nos traz para essa discussão o tempo inteiro, no sentido eu não vivo só, eu vivo em conjunto, dentro de um sistema onde se tem direitos e deveres estabelecidos, entre pessoas com as suas diversidades e chegar a um senso de negociação e consonante com, e respeitando o limite do outro, não é nada fácil. A gente sabe o quanto isso é difícil e é necessário que nós tenhamos condição e embasamento através das leis que regem a nossa sobrevivência digamos assim nas sociedades que sejam o condutor de toda a orientação daquilo que nós podemos avançar e daquilo que nós temos que ter sob controle, para que a gente não invada o espaço é um direito do outro. E como a internet ela amplifica todas as relações e essas relações envolve a relação pessoal a relação humana, a relação de negócio tudo isso vivendo em um mesmo... “é como se a gente tivesse na vida, o contato social e a vida virtual que é a relação dentro de um serviço que nos faz hoje ter acesso à informação que está lá do outro lado do mundo. Então fazer tudo isso chegar a uma congruência onde eu possa sobreviver e sem atingir e até ocupar o direito do outro, foram necessárias várias discussões que se chegou ao Marco Civil da Internet em 2014 e que gera um embasamento para a lei. Então quando vocês abrem o Marco Civil vocês vão perceber que dentro das disciplinas do uso da Internet vão ter princípios que eles estão citados novamente na lei. Então há o Marco e a discussão para a criação da lei que formalmente foi estabelecida em 2018. Algo muito recente. Então lá prevê a disciplina do uso da internet garantindo a liberdade de expressão de comunicação a manifestação do pensamento mas prevê também a privacidade e a proteção daquele que utiliza do meio,

desse meio de comunicação e convivência que é a internet.
Ela também prevê que a proteção de seus dados pessoais e a preservação da garantia da neutralidade nas redes e a gente sabe que hoje a rede social ela amplifica muito isso. Até pouco tempo atrás os meios de comunicação que nós tínhamos era e realmente eram o telefone era a carta que evoluiu para o telefone que evoluiu para o mensager. é aí que veio o Orkut, pensa na evolução de tudo isso até chegar agora no Instagram, no whatassap, que nós temos de forma rápida como contacto com o mundo inteiro, foi uma forma acelerar de nós termos acesso à informação de quase todo mundo exceto aqueles países onde a internet não é liberada, se tem a intranet, a Coreia do norte salvo engano, que utiliza esse serviço pensando inclusive na questão da privacidade das informações do país, mas que nós temos dentro da nossa realidade a amplificação do nosso relacionamento como um todo, então esse marco ela foi essa discussão uma discussão onde prevê inclusive a relação do homem com o meio e homem com o outro homem, e do homem com o meio ambiente porque nós sabemos que a utilização desse meio também em algumas situações ela poderia violar a questão do meio ambiente por conta da sua forma de amplificação que é através das antenas, satélites. Então esse marco ele vem para realmente gerar a lei, tem uma atualização inclusive em 2019, dessa lei, sobre o que tange ao direito do uso mas também os deveres daquilo que nós temos que ter como cuidado ao utilizar desse serviço. Você quer acrescentar alguma coisa antes de liberar o vídeo Apresentador 1?

Apresentador 1.

Não, é isso mesmo. E na verdade com relação a um marco civil é só fazer uma pontuação de curiosidade , o Marco Civil da Internet ele começou como a discussão se não me engano 2009. A proposta era que é. Como é que eu vou dizer ,que ele fosse discutido dentro a própria internet, e no final o resultado em 2014 acabou não agradando quase ninguém, porque as discussões na internet e segundo estudo que fizeram depois inclusive não gerou o documento que foi aprovado.
Fizeram ele discussões pontuar um monte de coisa colocar um documento ou outra coisa e associações elas são relacionadas a grupos estruturados do Direito como e não de lei da ordem. Mas eu me lembro que a Associação Nacional dos Delegados apresentou a manifestação contrária ao Marco Civil da Internet falando que o Marco Civil dificultaria ações de investigação uma vez que colocava é uma proteção excessiva aos direitos individuais dificultando inclusive, que eles fizessem investigações e uso de dados de telefone celular e meio de contatos telefônicos.
Isso gerou uma polêmica grande. Depois a Lei Geral, ela veio pra gente cita e logo em seguida. Ela veio para pontuar uma tentativa de correção e em cima do Marco Civil da Internet. E aí duas coisas né, o governo do Michel Temer governo houve proposta de criação de um órgão regulador de dados de modo que ele iria monitorar as empresas que faziam oferta de dados sobre inclusive exposição desses dados ao período.

E isso foi abortado não foi criado e logo o governo atual fez uma imposição de sanções inclusive com multa para as empresas no sentido de que ao solicitar, ao ser solicitado a acessar dados a empresa não poderia demorar mais do seis meses para liberar o acesso. E essa legislação já é uma realidade no Brasil levando em consideração esses dados que

eles não ainda que criptografados eles fiquem disponíveis em algum lugar para fins inclusive de legislação e a partir daqui a gente como planeja a falar sobre a lei geral de proteção dos dados, a gente acabou fala mais mais dela no decorrer da apresentação do que do Marco Civil, mas se a gente pode até por conta do avançado da hora, falar um pouco sobre o artigo, para gente ter um tempo também hábil para mandar o pessoal para discutir em cima do artigo e retomar com o preenchimento do formulário, pode ser assim? Vamos lá então. O artigo. Pessoal vocês estão conseguindo ver a projeção?

Apresentador 1

• 2:11:26 - 2:12:01
O artigo que nós escolhemos havíamos escolhido. Ele se chama: Ética de rastreamento de contato instantâneo usando aplicativos de telefone celular um controle da pandemia. Essa é uma tradução livre que a gente fez do inglês e foi publicada em Medical Journal of Medical éticos e eu pego o texto com o título em inglês a ética oficial que não se entende se compete completo e uso o Mobile Phone.
• 2:12:02 - 2:12:50

E4. Vamos lá, o artigo ele faz uma série de construções de conjunturas, leva em consideração o período de início da pandemia, foi publicado em maio de 2015, do que seria a tramitação para a execução e otimização de aplicativos de rastreamento. Como a gente viu nos vídeos, essa realidade ela acabou sendo sedimentada, ela acabou acontecendo de alguma forma mundo afora, mas ela precede essa iniciativa de alguma maneira pontua questões éticas que deveriam ser levadas em consideração as éticas e biomédicas e deveriam ser levadas em consideração em decorrência de um processo de implantação de uso de aplicativos de rastreamento. Eu fiquei também com temor de como isso vai afetar o meu trabalho. Cuido de pessoas. Tenho equipe com muita gente que eu tenho que falar, que eu tenho que direcionar... Aí, vem uma situação em que a pessoa se sente invadida por alguma coisa que foi determinada a partir dessa porteira aberta do uso dos aplicativos. Eu não sei como vai ser o futuro da telemedicina, por exemplo.

• 2:12:51 - 2:12:51

• 2:12:58 - 2:13:28
ele considera que o próprio rastreamento é uma prática muito antiga na saúde pública e que considera os contatos, todas essas questões que nós mostramos pra vocês no início da aula, a proporção de transmissão pré sintomáticos mostrou que os métodos atuais de rastreamento eram lentos, definitivamente. Vários países ao redor do mundo acabaram desenvolvendo aplicativos de rastreamento instantâneo de contatos.
Esses aplicativos eles vão realizar um mapeamento de eventos de proximidade. Como a gente mostrou e fornece alerta indivíduos que tiveram contato com a pessoa com à

infecção, e aí isso seria uma porta para o distanciamento físico inteligente e vai gerar essas questões da bioética que precisam ser discutidas. Na introdução ele vai pontuar o seguinte, até a confirmação oficial do surto e um bloqueio inicial na China, estima se que 5 bilhões de pessoas saíram da região de Omã, antes que o governo conseguisse fazer qualquer coisa. Essas pessoas se espalharam pelo continente asiático inteiro, fazendo uma propagação. O artigo traz a informação da facilidade de migração para o continente europeu, considerando voos diários e deslocamentos, os mais diversos.
O sucesso da diminuição desses casos na China se deve à velocidade de celulares ligados a programas de testagem intensivo. No artigo eles falam que não existe a possibilidade de você só fazer uso de dados de celular sem que outras medidas de saúde coletiva sejam atreladas a ele, e um desafio é transferir essa abordagem para diferentes países com diferentes culturas e diferentes regimes jurídicos.

As questões éticas são abordadas: 11 questões que precisam ser levadas em consideração no caso de implantação de aplicativos de rastreamento.

Com relação a benefícios e danos dessa implantação, fala que o fato da pandemia, ela tem um potencial para matar muitas pessoas e adoecer um outro número gigante de pessoas, vai tratar de acesso a serviços de saúde sobrecarga de serviços de saúde e que numa visão de barganha com as populações parece razoável se perder um pouco da condição até subjetiva do sigilo por conta de um bem maior que é evitar a morte de outras pessoas.
E aí fala lá: nem é preciso dizer que salvar vidas e reduzir o sofrimento é de imensa importância moral e há fortes razões para apoiar os esforços nesse sentido. Avaliação ética capaz de contribuir para o enfrentamento desses agravos precisa ser compreendida e analisada à luz da dramática escala de mortes e sofrimentos apresentados por esses dados, então esse é o benefício, e o dano seria as mortes que seriam causadas por conta disso.

Só que o distanciamento social inteligente e não inteligente, o artigo pontuou aqui no período da redação do artigo pontua que um terço da população estava vivendo em bloqueio ou restrições impostas por governos, e o que é necessário, que é social e culturalmente apropriado, vai ser diferente no mundo inteiro, então independente, você tem uma dificuldade maior de implementar medidas que sejam mais impositivas pelo poder repressor do Estado. A emergência de saúde pública ela vai trazer ações que são possíveis dada a situação que se estabeleceram e a mera existência de emergência não vai legitimar essas intromissões e a invasão da privacidade individual.
Não é só conscientizar as pessoas de que existe um problema, a pessoa precisa entender que há a restrição de movimento ela pode ser interrompida se todo mundo liberar o rastreamento de celular. Vamos a seguir as pessoas que forem identificadas vão por outro tipo de distanciamento social como o já citado aqui distanciamento vertical, que foi falado e isso geraria uma liberação para atendimento de outras questões, com o professor falou de questões econômicas e políticas e talvez por isso as pessoas se sentiriam impelidas a aceitar isso.
Com relação à privacidade o artigo pontua que antes da pandemia as pessoas já se preocupavam com a segurança de dados e privacidade, esse assunto não é novo e em combinação com essas medidas restritivas o compartilhamento de dados e rastreamento e ainda que de forma randomizada gera preocupação, e duas perguntas vão emergir

dessa questão da privacidade. Qual é a natureza dessa invasão de privacidade? Então, vão ficar sabendo o quê meu respeito? é a 2 pergunta é essa: invasão de privacidade pode ser justificada pela pandemia?
E o artigo fala que você trabalha com uma perspectiva de dois tipos de pessoas como se existisse uma gradação de zero a 100, a pessoa “A” que estaria numa ponta nessa régua, seria uma pessoa que se preocupa totalmente com a sua privacidade e quer manter total sigilo sobre suas informações, e a pessoa “Z” não se importa nem um pouco com isso. E o artigo pontua que as pessoas “Z” são em maior quantidade, e se não existem mais pessoas “Z” do que pessoas “A”, então esse tipo de iniciativa teria um relativo sucesso, porque a maioria das pessoas não se importa desesperadamente com o ser rastreada via satélite em deslocamentos, dentro das cidades ou por onde ela for, e a pandemia justificaria essa perda de privacidade desde que se prove que esse método de rastreamento é mais eficaz do que o outro método. Lembrem se que a gente está falando de maio de 2020.
O debate em torno de qualquer impacto sobre a privacidade, ele vai ser minimizado, altos padrões de segurança proteção e supervisão de dados vão está em vigor, seja por acordos nacionais como já mostrei o Marco Civil da Internet ou por acordos internacionais e que haveria transparência no uso de dados.

Apresentador 2

só para poder também exemplificar aqui dentro da lei está previsto que o tratamento de dados pessoais sensíveis em caso de bem maior com uma saúde de proteção à vida, incompatibilidade física do titular, o parceiro pode ser disponível, quer a tutela da saúde exclusivamente em procedimento realizado por profissionais de saúde serviços de saúde ou autoridade sanitária também estar autorizado, e tem uma convenção e uma redação dentro dessa lei prevendo assim o acesso à informação.

Apresentador 1

É assim onde a gente encontra discussão sobre possíveis conflitos entre liberdade e a privacidade, o artigo colocou a questão, é razoável manter um bloqueio de circulação de um país inteiro apenas para manter a privacidade das pessoas? Me parece que a resposta da maioria seria não. A gente pode sim, perder um pouco de privacidade para que as pessoas consigam continuar trabalhando, continuar produzindo, não necessariamente ficar em isolamento domiciliar como foi feito aqui, enfim, aqui no Brasil a gente pode dizer que foi feito com pouca rigidez, porque o pessoal fez muita festa em casa. O aplicativo pode ainda significar um aumento de liberdade uma vez que ele tira a pessoa do confinamento, e dar quem sai de casa a possibilidade de escolher se vai correr esse risco, e “n” questões ,e aí a pergunta: O aplicativo deve ser obrigatório? Aí é uma questão que a gente falou no início eu fiquei doido para falar isso mas deixei pra na hora que chegar da fala do artigo.
Pelo tipo de estudo que estava sendo feito na Inglaterra, no período, é 50 por cento da população instalando o aplicativo no celular, o aplicativo de rastreamento já um efeito positivo, no entanto se a gente tiver um número menor do que isso de pessoas instalando, os dados,eles não têm necessariamente a confiabilidade que deveriam ter. Se a pessoa próximas também está ali significa uma proteção maior para o usuário no caso de um contato com um individuo contaminado. Pode ser utilizado incentivo de uso, é uma sugestão até, caso a adesão seja baixa, tipo, dar recarga de celular gratuito para

quem instalar o aplicativo, e a pessoa que instala e pode doar “x” para instituição de caridade, para tentar estimular, mais essa medida é excludente, porque ela exclui quem não tem um celular apropriado para isso, por exemplo.

As responsabilidade que a foi o que o professor falou, isso tem que ter um cuidado muito maior ainda com os nossos, é, a palavra que me vem são desvalidos, mas sei lá, também acho meio pejorativo, mas é necessário ter que pensar nessas questões, é a pessoa que não tem celular? Na verdade as responsabilidades das instituições e dos profissionais, o artigo fala aqui para pessoas responsáveis por locais com aglomeração de pessoas ficava a responsabilidade de controlar o acesso, que é o que a gente tenta fazer hoje em dia mais ou menos no Brasil, aqui em Araçuaí a gente está fazendo, e monitorar a entrada e saída das pessoas dentro do comércio com o número de lotação pregado na porta desse comércio, e, é de responsabilidade do dono do comércio controlar o número de pessoas circulando lá dentro.

Os aplicativos móveis eles facilitariam isso e haveria uma boa razão para supor que a maioria das pessoas gostaria de sair do bloqueio e saber disso quando fosse trabalhar, ou fosse em uma lanchonete, saber que está ali seguro, ainda que subjetivamente, e poderiam contribuir para uma segurança uma sensação de segurança coletiva. Aí ele fala também sobre a ética de gerenciar a emergência do bloqueio, se for possível demonstrar que o aplicativo oferece o potencial de fornecer informações para permitir que indivíduos e administradores de instituições garantam uma saída inteligente e segura do bloqueio, há razão para o uso.

Sugere a necessidade de uma análise, aprofundar mais as questões éticas relacionadas ao processo de sair de um bloqueio, é um lockdown ou algo do tipo.

É potencialmente em uma série de bloqueios que fossem periódicos, em vez de fazer um bloqueio total, fecharam no final de semana, e sem que isso tivesse um impacto tão grande na vida das pessoas. E aí vem aquela pergunta que nós suscitamos aí com vocês, os dados devem ser apagados no final da pandemia?
E o artigo considera que uma maneira de aumentar a chance das pessoas aderirem é permitir que ela reúne dados de proximidade pode ser o compromisso da extinção desses dados, depois da pandemia. Os dados que ele produz podem ser recursos inestimáveis para proteção de gerações futuras de danos graves ou seja o meio de pesquisa desenvolvimento de método de modelagem e avaliação, então esses dados eles também estão a realizar se você não pode só prometer para as pessoas não pagar.
Isso pode servir como objeto de estudo para aperfeiçoamento dessa técnica no futuro. Então se esses dados forem retidos para este tipo de uso é uma série de questões importantes relacionadas à segurança, supervisão, e propriedades, precisarão de respostas claras e aplicáveis. E aí, confiança e segurança pública bem fundamentadas e além de provisão de respostas claramente justificadas e articuladas as perguntas, que foram feitas aí acima, os requisitos provavelmente vão incluir perguntas que digam respeito a estabelecimento de mecanismos eficazes e transparentes, supervisão responsável inclusive dos dados, e a criação de um órgão de supervisão de ética ,incluindo membros públicos, de pessoas da comunidade ou estudiosos da área, pesquisadores, pactuação e publicação desde o início dos princípios éticos que orientaram o uso desses aplicativos, uso um algoritmo transparente, auditado e de fácil

explicação e com altos padrões de segurança de dados e proteção eficaz em torno do uso da propriedade dos dados, reservando o direito individual que inclusive está no nosso Marco Civil da Internet. E aí ,tem a questão da equidade, igualdade e justiça que fala que todas as emergências de saúde pública levantam questões relacionadas a isso. Essas situações elas comumente ocorrem infrações à Justiça e discriminação e estigma.
A resposta ao covid não foi diferente de outras emergências de saúde pública, e a grande questão em debate sobre o uso de aplicativos é um reconhecimento da importância dele e se ele envolve relações sérias relacionadas à equidade e justiça. E aí por último ele fala sobre consistência e comparação de casos. Se for comprovado que o uso do rastreamento de contato combinado com testes generalizados como foi feito lá na China é eficaz, surgiram questões sobre a implicação ética de seu uso em outras doenças, especialmente em surtos de doenças infecto contagiosas. É aceitável ou mesmo obrigatório, que o aplicativo projetado especificamente para ser usado para o covid, possa ser aplicado imediatamente ao caso da gripe sazonal,por exemplo?Isso aí é uma questão que precisaria ser levada em consideração. A gente não pode naturalizar o uso numa situação de pandemia drástica, naturalizar isso e aplicar imediatamente outras lógicas. Então há uma preocupação com relação a isso, embora existam diferenças também existem semelhanças, moralmente significativas entre a covid e a gripe sazonal, e entre outras doenças.

Como conclusão o artigo fala que há evidências científicas e epidemiológica sugerem que um aplicativo desse tipo tem o potencial de contribuir para reduzir o sofrimento causado pela pandemia e pelo confinamento, e minimizar os danos, causados pelo período do bloqueio.

E5

Você falando desse monitoramento do vídeo, lembrei que a gente teve algo também assim, Não sei exatamente se foi desse jeito, com a polícia lá olhando, mas em Montes Claros tinha uma pulseirinha amarela né, não sei se ainda estão usando, mas no ano passado as pessoas que vinham de outras cidades recebiam uma pulseira amarela que identificava que essa pessoa que ficar em isolamento, não podia ficar andando por aí. É um recurso não tecnológico, mas tem a mesma sensação de saber que se uma pessoa usa a pulseirinha amarela, ela tem que está em casa. Vendo essas explanações, fico receosa sobre o que pode acontecer com pessoas que procuram o serviço de saúde, por exemplo.

E2: Eu só queria deixar três perguntas que não precisam ser respondidas na perspectiva seguinte. Nós estamos trabalhando aqui na lógica de responsabilidade para com um bem coletivo e visando entre o qual o indivíduo deve se sobressair e no comparativo, o coletivo sempre vai ganhar.
Porém não à custa da anulação do indivíduo. Mas a pergunta é que a gente está dentro da situação vivida no nosso caso específico de uma pandemia o a deixar três perguntas preventivas, bem preventivas, antes da pandemia: De onde surgem as epidemias? Como prevenir as epidemias?
Então teria que trabalhar a perspectiva ética de estudo nesse sentido para evitar que outras epidemias venham, e a gente não ficar sofrendo o tempo inteiro restringindo

liberdades e se for possível a gente trabalhar de uma forma preventiva, e eu creio que há possibilidade de fazer isso.

Nós sabemos aí a relação das doenças infecto contagiosas que é um triângulo entre o agente, o vírus hospedeiro, a pessoa e o ambiente.

As três perguntas estão mais relacionadas ao ambiente aos hospedeiros. Então como agente prevenir é ir trabalhando com um ambiente na perspectiva de evitar que surjam ou que os agentes se tornam o ganho de virulência suficiente para causar doenças,
E o outro tripé dessa perspectiva é como somos e podemos melhorar nossa capacidade de defesa, então são questões que estão dentro do ecossistema de visão ecológica, uma visão mais ampliada que ela não deixa de ser uma questão bioética e a gente não pode esquecer disto, esse vírus, essa pandemia não caiu do céu, de uma certa forma nós trabalhamos para que ela existisse e que ela esteja da forma como está.
Não podemos preocupar somente em controlar, eu não vou dizer eliminar igual muitas às vezes a gente escuta, combater, matar, acabar o vírus, isso não vai acontecer. A gente tem que buscar o equilíbrio e moderação, isso em tudo. Então são essas perguntas que ficam aí, se tiverem um ou dois comentários ficam livres se não seguimos a vontade. Não podemos também nos esquivar da responsabilidade profissional. Tanto de proteger o ser humano em sua integralidade como também para reservar à sociedade a condição da individualidade humana. Para mim a questão vai além das minhas preferências pessoais.

RESPOSTAS DOS QUESTIONÁRIOS Pós-TESTE

Questões:

Questão 1. Em casos de emergência de saúde pública, é correto que o direito ao sigilo e ao anonimato sejam restringidos? (Justifique sua resposta)

Questão 2. De que forma o rastreamento de pessoas fere liberdades individuais?

Questão 3. Você instalaria em seu dispositivo móvel um aplicativo que permitisse que mapeassem seus deslocamentos? (Justifique sua resposta)

E1

1. Não, A quebra de sigilo pode ser usado para um bem comum , não se deve restringi-los.
2. Fere a privacidade das pessoas, a liberdade de deslocamentos.
3. Não, devido a segurança dos sistemas de hoje serem as vezes invadidos por hackers e situações inesperadas poderiam acontecer.

E2

1. Não, as informações de onde estive podem ser usadas inclusive para que eu seja perseguido por alguém. Deve-se levar em contato a fragilidade do uso de

dados, uma vez que hoje em dia acontecem muitos casos de vazamentos que expõem as pessoas.
2. Na medida que discrimina e restringe o direito de ir e vir.
3. Sim com a condicao de bloquear quando julgar necessario e obedecendo pactuacao prévia. Não há mal em colaborar.

E3

1. Sim, pois passa a prevalecer um direito maior, que o combate em favor da vida.
2. Podem impactar diretamente na quebra da individualidade, do oculto do ser, no entanto deve ser usando de forma criteriosa, restrita, como nos casos de pandemia.
3. Sim, desde que seja um aplicativo seguro, com limitações de dados assegurados, com finalidades claras, assegurados pela legislação de proteção de dados. Seguindo o modelo de proteção de dados de pacientes, que já existe.

E4

1. Não, pois não quero que informações obtidas do meu telefone fiquem disponíveis sem que eu saiba para que serão utilizadas.
2. Quando fere o princípio da dignidade humana e viola direitos fundamentais.
3. Depende da justificativa objetivando bem coletivo. Temo por mim e por meus pacientes.

E5

1. Em situações de emergência de saúde pública é aconselhada a adoção de medidas de rastreamento de pessoas doentes ou contatos para que seja possível o bloqueio e diminuição na transmissão de agentes causadores de doenças com potencial de morbidade/mortalidade importantes. Essa é inclusive uma prática recomendada pela OMS e aplicada desde muito cedo pela humanidade no controle de epidemias. Para tanto, o sigilo é de certa forma quebrado por meio de notificações compulsórias, porém, apesar dessa quebra deve ser mantida a segurança dos dados da pessoa por meio dos órgãos de vigilância, tendo em vista que deve ser assegurado ao doente o direito à não estigmatização e não incriminação. Não acho que dados pessoais devam ser disponibilizados sem que se saiba como serão de fato utilizados.
2. Rastrear poderia ferir direitos de ir e vir ou da privacidade/individualidade se não houvesse um rigor ético na obtenção e tratamento dos dados.
3. Permitiria desde que fosse seguidos os princípios éticos e de proteção de dados.

E6

1. Não. Mesmo frente aos casos de emergência em Saúde pública o sigilo e anonimato devem ser respeitados para serem cumpridos os preceitos éticos relacionados aos seres humanos.

2. A medida que é exposto de forma universal as informações dos indivíduos. Tem que delimitar até onde se pode intervir.
3. Não, porque também não quero que sejam refringidos os meus direitos em relação ao sigilo e anonimato.

E7

1. Sim e Não... Como foi discutido a importância de ter seus dados armazenados. E a preocupação do vazamento dos dados. É uma realidade sem volta, mas eu acho que não teria segurança nisso. Alguns países obrigarão as pessoas a usar, o que é errado.
2. Quando extravia os dados do individuo, quebrando o sigilo do direito dos humanos. Precisamos de regas claras
3. Sim... Mudei a minha opinião. Vou aderir sim, pois será de grande valia para meio proteger até adaptar ao convívio do vírus. Não vejo algo como que possamos lutar.

E8

1. Sim. Respeitando as liberdade e Individualidades das pessoas, mas sem prejudicar o interesse coletivo. Nesse caso, deve se levar em conta a atemporalidade da informações e ainda, sobre a proteção dos dados e punição em caso de descumprimento dessa proteção.
2. Fere direitos individuais universais. O rastreamento pode acolher as informações em excesso, além disso deve-se observar sobre a armazenagem e proteção das informações.
3. Sim. Se esse rastreamento for para colaborar, em benefício do bem coletivo, auxiliar nos casos de saúde pública. E tendo segurança na proteção dos dados.
