# Supplementary material for: The use of individual tracking programs in public health: a bioethics dilemma
Source: Rev Bras Enferm. 2024 Aug 19;77(Suppl 4):e20230041. doi: 10.1590/0034-7167-2023-0041 (PMC11338528; doi:10.1590/0034-7167-2023-0041)
Supplement: Supplementary file 3 [file 0034-7167-reben-77-s4-e20230041-suppl03.pdf]

DADOS SOBRE SEMINÁRIO DE BIOÉTICA E RASTREAMENTO DE INDIVÍDUOS NA SAÚDE

Pesquisador Responsável: Ricardo Jardim Neiva

Instituto Federal do Norte de Minas Gerais – Campus Araçuaí

Contato: ricardo.neiva@ifnmg.edu.br

Data da coleta de dados: 20/05/2021.

Conteúdo do material:

Arquivo I: anotacoes_analise_seminario – Contém as anotações realizadas a partir das respostas dos questionários aplicados antes e após o seminário temático sobre rastreamento de indivíduos e seus dilemas na bioética. Corresponde ao primeiro e terceiro momentos do seminário.

Arquivo II – transcricao_audios_seminario_bioetica – Contém a íntegra das respostas dos questionários aplicados antes e após os seminário temático sobre rastreamento de indivíduos e bioética, bem como a transcrição de todo o seminário. Corresponde ao segundo momento do seminário.

Coleta dos dados: A coleta se deu através da aplicação de questionário em formulário do Google Forms ® e gravação do seminário em dispositivo de captura de áudio e vídeo para posterior registro em forma de transcrição.
