# Supplementary material for: The use of individual tracking programs in public health: a bioethics dilemma
Source: Rev Bras Enferm. 2024 Aug 19;77(Suppl 4):e20230041. doi: 10.1590/0034-7167-2023-0041 (PMC11338528; doi:10.1590/0034-7167-2023-0041)
Supplement: Translation

# Translation: O uso de programas de rastreamento individual na saúde pública: um dilema da bioética

**DOI:** 10.1590/0034-7167-2023-0041pt

**Authors:** Ricardo Jardim Neiva, João Felício Rodrigues, Ana Paula Oliveira Nascimento Alves, Patrícia de Sousa Fernandes Queiroz, Leila das Graças Siqueira, Clara Cynthia Melo e Lima, Francisca Souza Santos Dias, Renê Ferreira da Silva-Júnior

**Affiliations:** I Instituto Federal do Norte de Minas Gerais. Araçuaí, Minas Gerais. Brasil; II Universidade Estadual de Montes Claros. Montes Claros, Minas Gerais. Brasil; III Instituto Federal do Norte de Minas Gerais. Januária, Minas Gerais. Brasil; IV Instituto Federal do Sul de Minas Gerais. Machado, Minas Gerais. Brasil

**Keywords:** Bioética, Programas de Rastreamento, Saúde Pública, Revisão Ética, Universidades

## Abstract

**Objetivo:**

compreender as perspectivas bioéticas no uso de dispositivos móveis de rastreamento.

**Métodos:**

estudo teórico fundamentado através de pesquisa-ação, realizado com oito pós-graduandos de uma universidade pública. Utilizou-se grupo focal, com metodologia de análise de conteúdo temática com estrutura de *codebook*, aprovado pelo Comitê de Ética em Pesquisa.

**Resultados:**

a partir da análise, constatou-se uma preocupação sobre o uso dos dispositivos findada a pandemia. O uso ou não do dispositivo, direitos inerentes à pessoa humana, legislação e efetividade dos métodos aprofundam interpretações, passando os participantes de uma concepção personalista do tema a uma visão voltada a implicações profissionais sobre os métodos.

**Considerações finais:**

o debate sobre o impacto do uso de dispositivos tecnológicos na saúde, em especial os que impliquem restrição de direitos que se referem à vida privada do indivíduo, passa por uma discussão de caráter profissional, além da exigência de claro regramento sobre o tema.

## INTRODUÇÃO

A bioética é uma área de estudo e conhecimento que busca, acima de outros conceitos, a defesa da vida, do meio ambiente e da saúde. Para tanto, vale-se de sua estrutura academicista, plural e não corporativa, focando em questões sensíveis à vida humana. O termo transmuta-se com o passar do tempo em um meio de comunicação de várias áreas do conhecimento, em questões morais, da ética, religiosas, técnico-científicas e outras mais, com a intenção de traçar estratégias para a proteção dos indivíduos^(1-2)^.

No percurso histórico da bioética na lógica principialista, a sociedade propõe as discussões da área em temas, como autonomia, beneficência, não maleficência e justiça^(3)^. Por conseguinte, deve-se ressaltar que a versão intervencionista engloba áreas como a geopolítica, emergência e reemergências globais, equidade e igualdade, entre outras. A bioética também versa nas ciências humanas, em especial a sociologia, em temas como “integridade epistemológica das ciências da saúde, análises ético-políticas em torno do acesso e segurança de novas e velhas tecnologias em saúde”^(4)^.

Na evolução da assistência em saúde, é necessário realizar reflexão constante sobre o uso de dispositivos tecnológicos para atendimento de demandas estruturais, de forma que não haja influência negativa e/ou prejuízos para a coletividade. A produção e o uso de dados em tecnologias digitais seguem em avanço a partir da segunda década do século XXI, aumentado a partir da pandemia de covid-19^(5-7)^. Modelagens computacionais e o uso de algoritmos proporcionam uma nova forma de produção do conhecimento, que é cada vez mais utilizada. Dados ganharam relevância para relações pessoais, comerciais, econômicas, políticas e sociais. Faz-se necessário que informações de boa qualidade estejam disponíveis a equipes profissionais para análise básica de dados epidemiológicos e orientem a produção de informações e medidas clínicas de diagnóstico, manejo, além da reabilitação e prevenção de novos casos^(8)^.

Pela inexistência de perspectiva de bloqueio vacinal de larga escala nos meses iniciais da pandemia, empresas de *software* iniciaram o trabalho de aperfeiçoamento das ferramentas para o caso específico da identificação de contatos da doença. Essa é uma nova modalidade de controle epidemiológico, embora aspectos de suas aplicabilidades ainda não tenham sido devidamente analisados^(9-11)^.

No Brasil, o aplicativo utilizado foi o *API Exposure Notification*, disponibilizado gratuitamente pelo Ministério da Saúde em parceria com as empresas de tecnologia *Apple* ^®^ e *Google* ^®^. O mesmo entrou em vigor em agosto de 2021 na plataforma Coronavírus-SUS. Em 2021, foi publicada cartilha que atenta para a regulamentação do uso de dados individuais em dispositivos de rastreamento^(12)^. O documento busca compatibilizar a regulamentação de proteção de dados e o monitoramento individual com interesses públicos e manutenção da segurança jurídica. A Lei Geral de Proteção de Dados Individuais, sancionada em 2018, deve, portanto, ser aplicada à lógica de dados individuais na pandemia. A tecnologia utilizada no Brasil seria de descentralização de dados, ficando os arquivos reservados ao aparelho da pessoa que instalou o aplicativo^(13)^. No mesmo passo, a Portaria nº 2358, de 2 de setembro de 2020, cria incentivos financeiros e operacionais à dinamização do rastreio e monitoramento de contatos^(14)^. Isso permite que municípios criem de maneira autônoma sistemas de informação para registros de rastreamento, desde que em interoperacionalidade com o sistema federal e-SUS Notifica. Após o período crítico da pandemia, estudos demonstram que o sistema sincronizou compulsoriamente dados de casos suspeitos de covid-19, com elevado número de informações incompletas e tardias para ações efetivas^(5)^. Tal ato coloca em risco conceitos de segurança de dados, direito ao anonimato e privacidade. Retomando à base principialista da bioética no Brasil, há um flagrante conflito dos atos sobre a autonomia, não maleficência e justiça.

A vigilância e o monitoramento em saúde são práticas exercidas pelo estado democrático e garantidas por leis que separam o interesse individual do coletivo. A covid-19, entretanto, promoveu uma colisão entre o direito à privacidade e direitos fundamentais à saúde e à vida, necessitando que sejam estabelecidas balizas de ponderação^(5,15)^.

O uso de tecnologias em rastreamento cruza a bioética em dimensões que vão do pessoal ao coletivo. É um dos novos paradigmas da área, abordado também como um dos possíveis usos de Inteligência Artificial (IA), tendo entre eles a engenharia genética e a telemedicina^(15)^. Diante do exposto, busca-se compreender o entendimento pessoal dos pós-graduandos de uma instituição pública atuantes na área da saúde acerca das questões bioéticas envolvendo o uso de dispositivos de rastreamento. Os aspectos bioéticos envolvem o monitoramento pessoal, com restrições de liberdades individuais em benefício de uma coletividade em situação de epidemia com risco de morte.

O tema em questão não apresenta um consenso global, mas direciona elementos norteadores do pensamento bioético. Entre eles, podemos destacar o conflito entre a privacidade e a liberdade individual, obrigatoriedades envolvidas e as responsabilidades pessoais e institucionais sobre o processo^(16)^. Portanto, a bioética encampa discussões sobre a amplitude e implicações do monitoramento de pessoas através de tecnologias, com argumento de preservação da saúde coletiva.

## OBJETIVO

Compreender o entendimento pessoal sobre as perspectivas bioéticas envolvidas no uso pessoal e profissional de dispositivos móveis de rastreamento.

## MÉTODOS

### Aspectos éticos

A pesquisa considerou as diretrizes da Resolução n^o^ 510, de 7 de abril de 2016, e Resolução n^o^ 466, do Conselho Nacional de Saúde, de 12 de dezembro de 2012. O Termo de Consentimento Livre e Esclarecido (TCLE) foi assinado por todos os participantes e pesquisador responsável. O projeto foi aprovado sob parecer do Conselho de Ética em Pesquisa da Universidade Estadual de Montes Claros, anexado à presente submissão. As transcrições foram elaboradas utilizando P1, P2, P3... (“P” para o participante e numeração de acordo com o preenchimento dos questionários, em sequência), de forma a garantir o anonimato dos participantes.

### Tipo de estudo

Trata-se de estudo qualitativo, baseada na *COnsolidated criteria for REporting Qualitative research* (COREQ), que utiliza como fundamentação teórica a pesquisa-ação e metodologia de análise aplicada de análise temática em suas etapas^(17-18)^. A construção da análise ancora-se na perspectiva de elaboração de relatório utilizando a mesma metodologia, que posteriormente foi revisto junto com todos os registros sob o guarda-chuva teórico da perspectiva bioética.

### Procedimentos metodológicos

A coleta dos dados ocorreu durante o evento do seminário temático, através de análise dos formulários e gravação deste, com intervenções dos participantes. Foi dividida em três momentos:

- Primeiro momento: inicia-se com um questionário acessado pelos participantes na plataforma *Google Forms* ^®^, contendo cinco questões, a saber: nome completo; e-mail; em casos de emergência de saúde pública, é correto que o direito ao sigilo e o anonimato sejam restringidos? (Justifique sua resposta); de que forma o rastreamento de pessoas fere liberdades individuais?; você instalaria em seu dispositivo móvel um aplicativo que permitisse que mapeassem seus deslocamentos? (Justifique sua resposta);
- Segundo momento: apresentação do seminário temático, com gravação do conteúdo das discussões na plataforma *Google Meet* ^®^, orientado a partir de bibliografia que considera implicações éticas dos dispositivos de rastreamento relacionadas a benefícios e danos, distanciamento físico inteligente, conflitos entre liberdade e privacidade, obrigatoriedade do uso dos aplicativos e responsabilidades institucionais e profissionais^(16)^;
- Terceiro momento: repetição do questionário após as discussões com as mesmas questões respondidas antes do início dos trabalhos.

Após a transcrição das falas e intervenções durante o seminário, o material foi separado e catalogado para sistematização da análise.

### Cenário do estudo

A pesquisa foi realizada em um programa de pós-graduação *stricto sensu* de uma universidade pública no estado de Minas Gerais.

### Coleta e organização dos dados

O estudo ambientou-se em apresentação de seminário temático da disciplina de Bioética, utilizando ambiente virtual *Google Meet* ^®^ para gravação da discussão e plataforma *Google Forms* ^®^ para coletar as respostas dos participantes sobre o tema no primeiro e terceiro momentos.

Os participantes foram profissionais da área de enfermagem, medicina, odontologia e nutrição e ciências contábeis com auditoria em saúde, em grupo focal realizado em encontro único. Utilizou-se, portanto, critério não probabilístico, ocorrido por conveniência ou acesso^(19)^. Os critérios de inclusão utilizados foram estar matriculado no programa e na disciplina de Bioética ou ser professor da mesma no primeiro semestre de 2021 e ter participado da aula Conflitos Bioéticos do Uso de Dispositivos de Rastreamento de Indivíduos.

Os dados foram coletados da seguinte forma: o primeiro momento compreende a resposta do questionário na plataforma; o segundo momento compreende a exposição dialogada do tema no ambiente virtual; e o terceiro momento compreende nova coleta de informações, também em plataforma.

### Análise dos dados

Os dados coletados seguiram etapas de pré-análise com imersão de leitura, exploração do material, codificação e tratamento dos resultados obtidos^(18)^. As informações contidas levaram em conta: a) coesão semântica; b) criação dos códigos, considerando as características dos dados mais relevantes e definição conceitual de cada código; c) pesquisa de temas, agrupando os códigos em temas emergidos e revisão dos temas pelos avaliadores e preparação do mapa temático; d) definição dos temas em pactuação de cada tema em separado; e) elaboração do relatório com seleção de exemplos de trechos das participações e observações. Foram categorizadas na análise questões elencadas, tais como liberdade individual e responsabilidade coletiva, direito ao sigilo e anonimato, demanda espontânea, restrições da disponibilização dos dados, tempo de duração dos efeitos da intervenção, implicações em práticas profissionais na saúde. Essas foram sintetizadas em três eixos temáticos, tais como direito ao sigilo e anonimato, das liberdades individuais e pessoalidade da escolha e demanda espontânea por aplicativos de rastreamento individual.

## RESULTADOS

Como sistematização dos temas identificados a partir de análise temática do material coletado, foram feitos registros de forma a contemplar as relações entre as discussões dos três momentos. Os elementos codificados relacionam-se entre os posicionamentos pessoais e profissionais dos participantes, a partir da intervenção proposta. Os resultados apresentados seguem a lógica estabelecida a partir do padrão da discussão.

### Pessoalidade no sigilo, anonimato e liberdades individuais pelo “bem comum”

No primeiro momento, os participantes apresentam argumentação que pressupõe que as preocupações sejam de entendimento da questão em perspectiva pessoal. Quando são questionados sobre a restrição do direito ao sigilo e anonimato em casos específicos de emergências em saúde, todos participantes concordam. Usou-se terminologia em primeira pessoa e afirmações que remetem a códigos como “bem comum”, “interesse coletivo”, “colaborar”, “entender” e “todo mundo”.

*Sim, eu penso que, para o uso de aplicativo no celular, eu teria o benefício de saber se tive contato com alguém contaminado. Hoje em dia, estamos todos nas ruas sem saber. Pode estar em qualquer lugar. Então, o interesse coletivo é isso: uns ajudando os outros.* (P3 - Primeiro momento)

*Sim, não veria problema. É uma atitude pelo bem comum, desde que todo mundo use. Assim, a Covid vai embora mais rápido.* (P4 - Primeiro momento)

*Sim, se a situação da necessidade coletiva exigir a ações que determinem a prevenção ou controle de doenças, por exemplo.* (P8 - Primeiro momento)

Sobre direitos de liberdade individual, os participantes sugerem, a princípio, que seja observado o direito de cada pessoa. No entanto, trazem discussões acerca da necessidade de colaboração coletiva em casos emergenciais. O mesmo pode ser dito quando inqueridos acerca de sua disposição em instalar um aplicativo de rastreamento de contatos em seu celular:

*Cada pessoa faz sua escolha. Eu instalaria, sim. Seria bom pra mim e bom para todos.* (P2 - Primeiro momento)

*Eu concordo em utilizar. Para controlar um mal como a Covid, vale o esforço. É um momento em que precisamos expressar solidariedade.* (P5 - Primeiro momento)

*Num entendimento global, acho que vale à pena. Ajudaria a identificar onde está se transmitindo a doença em questão para intervirmos.* (P8 - Primeiro momento)

### Debate no seminário: a verve profissional sobre a perspectiva da intervenção na vida pessoal

Durante o seminário, os relatos começam a se alternar enquanto são apresentados dados que envolvem a Lei Geral de Proteção de Dados^(13)^. Há uma ruptura da discussão entre o debate pessoal e acadêmico que imediatamente aponta para questões que dizem respeito à prática profissional. A relutância em abrir mão de algo em prol do bem comum migra para a contestação da segurança atual do uso de dados do paciente e outras experiências, como o prontuário eletrônico:

*Eu já trabalhei com dados de pacientes com doenças mais graves. O uso do SINAN, o informe para as centrais com o nome da pessoa, eu sempre entendi como seguro. Agora, a observação da pessoa pelo celular, eu tenho um grande pé atrás. Não precisa chegar a tanto.* (P3 - Segundo momento)

*Que você tenha a preservação de dados de pacientes apenas para pessoas que estejam ali. Quando a gente manda nossas fichas de notificação para a vigilância sanitária, estou de certa forma quebrando o sigilo da consulta. Mandam lá para vigilância para uma pessoa que não estava naquela consulta.* (P5 - Segundo momento)

*Acho que, mesmo em casos de emergência em saúde pública, devemos manter o sigilo e anonimato para cumprir os preceitos éticos relacionados aos seres humanos, né?* (P6- Segundo momento)

*Já falei que eu não instalaria* [...] *continuo com a mesma opinião. Até que me provem que funciona cem por cento e que todo mundo instalando faz a diferença na pandemia, eu não mudo de ideia, nem recomendo pros meus pacientes.* (P7 - Segundo momento)

No terceiro momento, a repetição das questões mostra uma inversão de tendência com aprofundamento dos condicionantes que antes foram sequer citados pelo grupo. Surgiram temas como exigência de tempo de duração do uso dos dados até que esses sejam deletados, menções a outras situações em que tenha havido a ruptura da proteção de dados por invasões por *hackers*. O receio da ruptura da relação entre profissional, equipe de saúde e usuário, em caso de falha do método, está contida nas falas que agora impõem condições de caráter técnico ou simplesmente repelem a iniciativa.

*Não, as informações de onde estive podem ser usadas inclusive para que eu seja perseguido por alguém. Deve-se levar em contato a fragilidade do uso de dados, uma vez que hoje em dia acontecem muitos casos de vazamentos que expõem as pessoas.* (P2 - Terceiro momento)

*Não, pois não quero que informações obtidas do meu telefone fiquem disponíveis sem que eu saiba para que serão utilizadas. Temo por mim e por meus pacientes.* (P4 - Terceiro momento)

*Em situações de emergência de saúde pública, é aconselhada a adoção de medidas de rastreamento de pessoas doentes ou contatos para que seja possível o bloqueio e diminuição na transmissão de agentes causadores de doenças com potencial de morbidade/mortalidade importantes. Essa é inclusive uma prática recomendada pela OMS e aplicada desde muito cedo pela humanidade no controle de epidemias. O sigilo é de certa forma quebrado por meio de notificações compulsórias, porém, apesar dessa quebra, deve ser mantida a segurança dos dados da pessoa por meio dos órgãos de vigilância, tendo em vista que deve ser assegurado ao doente o direito à não estigmatização e não incriminação. Não acho que dados pessoais devam ser disponibilizados sem que se saiba como serão de fato utilizados.* (P5 - Terceiro momento)

*É uma realidade sem volta, mas eu acho que não teria segurança nisso. Alguns países obrigarão as pessoas a usar, o que é errado.* (P7 - Terceiro momento)

## DISCUSSÃO

### Direito ao sigilo e anonimato

Em casos de emergência de saúde pública, é correto que o direito ao sigilo e o anonimato sejam restringidos? Na análise do conteúdo coletado no primeiro momento, é perceptível uma ênfase nas respostas a respeito de sigilo, não se repetindo quando referenciada ao “anonimato” presente na primeira questão. Há uma quebra de assertivas sobre o tema. Os participantes concordam que é possível se permitir uma invasão para o bem da coletividade de maneira personalista e sem imposição de condicionantes. O sigilo e o anonimato versam com a bioética na premissa de direitos inegociáveis que sustentam a autonomia do indivíduo^(16)^.

As exceções apontadas previamente seriam uma regulação a partir do poder do estado, de forma a assegurar que as informações sejam utilizadas para fins apenas científicos. Na Carta dos Direitos dos Usuários da Saúde, compreendem-se o sigilo e o anonimato como condições de proteção para o usuário dos serviços, lembrando que as ações desses devem prever resguardo dessas duas premissas em todas as etapas do processo de atendimento ou assistência^(20)^. Análise conduzida por Pagliari^(21)^ aponta que as grandes questões relacionadas ao tema são exatamente sobre sigilo, muito embora rastreamento de indivíduos para monitoramento seja prática considerada usual na área da saúde. Consideram-se peças-chave o anonimato, a segurança dos dados e o resguardo de direitos individuais que sejam próprios das sociedades livres.

Como fechamento das discussões relacionadas ao tema, houve várias menções no terceiro momento à sedimentação do entendimento coletivo, da noção de delimitação da interferência no direito individual no uso de tecnologias de rastreamento. Citações envolveram termos como “bem coletivo”, “bem de todos” e “segurança”.

Durante o segundo momento, coloca-se a condição do sigilo e do anonimato como fundamental para o rastreamento de pessoas, como exemplificado em P5 e P6. O argumento fundamental é de que o número de pessoas que não se importam em abrir mão de alguma forma de sua privacidade para o bem comum é muito maior do que o número das pessoas que se importam. Assim, desde que seja preservado um acordo com bases na democracia das nações e com regras bem definidas, a estratégia seria aceita^(16)^. Portanto, após a abordagem do questionário no terceiro momento, surge a necessidade de adequação de condicionantes, preservando a ética da autonomia.

Estudos recentes apontam que a modalidade de rastreamento de indivíduos não demonstra segurança sobre sua efetividade, considerando que os condicionantes apontados na pesquisa estejam relacionados e inexistindo uma iniciativa 100% segura^(5)^. Os aplicativos utilizados com maior adesão na Europa e América do Norte armazenam centralmente dados do usuário, o que os tornariam vulneráveis a ataques de *hackers*, quando armazenados no dispositivo móvel do indivíduo^(22)^.

Recentemente, o Supremo Tribunal Federal (STF) reafirmou em recurso extraordinário o direito do cidadão sobre seus dados pessoais e de saúde, em pleito onde o Ministério Público Federal solicitava ao Conselho Federal de Medicina a edição de resolução que autorizasse médicos, gestores e instituições a disponibilizar prontuários e demais informações sem autorização de pacientes ou de seus familiares^(23)^.

### Das liberdades individuais e pessoalidade da escolha

Para além da reflexão sobre o direito individual ao sigilo e anonimato de informações pessoais, é correto separar essa reflexão de outra, tão importante quanto, porém situando-se no âmbito do direito de ir e vir: a questão da liberdade. A pandemia de covid-19 teve como grande consequência mundial a restrição do deslocamento de pessoas. Em um mundo onde temos facilidade de deslocamento até intercontinental, é necessário analisar até quando é um direito do estado a restrição da liberdade individual.

Tal debate hoje está na dimensão pessoal e coletiva, estimulada de maneira belicosa por posicionamentos político-partidários. Como segunda questão do questionário, foi apresentado aos participantes: de que forma o rastreamento de pessoas fere liberdades individuais?

O bloqueio de deslocamentos afeta dramaticamente o direito de ir e vir. A ideia central discutida durante o seminário é a lógica do chamado benefício relativo. A restrição de liberdade é apontada como um encarceramento do qual as pessoas querem se livrar o mais rápido possível. Diante disso, a aceitação de uma ferramenta que restrinja o deslocamento por um período pequeno de tempo, com um vislumbre de liberação posterior, é bem aceito pela maioria, como em P4, P5 e P8. A visão destoaria, portanto, do poder restritivo do estado no cárcere, ou condições associadas ao crime.

Em debate sobre o compromisso social e coletivo, há dúvidas sobre os rumos tomados no Brasil que venham a ferir princípios básicos da ética principialista. Para os participantes, a limitação das liberdades individuais sustenta a percepção de que deve haver segurança individual, como citado pro P6 e P7, devendo ser fomentada pelo Estado. É um reflexo de tendências na pandemia de limitações em discussões sobre invasão de privacidade durante a pandemia nos Estados Unidos, com a busca ativa realizada com contatos após a primeira detecção de proximidade com caso de covid-19^(24)^. Governos de países como China, Coreia do Sul, Israel e Índia adotaram estratégias extremamente coercitivas no combate à covid-19^(21)^. Sutis questões bioéticas sustentam o posicionamento adotado após o seminário. Em regimes totalitários, há a obrigatoriedade, e em democracias estabelecidas, a judicialização da bioética para nortear as decisões^(24)^. Em especial na Coréia do Sul, foram usados dispositivos para “rastreamento preciso da localização de indivíduos identificáveis, mineração de dados pessoais, tecnologia de reconhecimento facial abrangente e dispositivos de contato direto com o corpo para fazer cumprir a quarentena”^(25)^. A categoria a seguir reforça a percepção de necessidade de controle pessoal ou segurança dos dados.

### Demanda espontânea por aplicativos de rastreamento individual

De acordo com estudos de Parker ^(16)^, a experiência particularizada de se colocar ao dispor do estado para difundir informações de cunho pessoal é relativa, sendo também um preço a ser pago apenas com regras claras e sob pretexto de que se traga algum alento ou segurança posteriormente. A questão é: você instalaria em seu celular um aplicativo que permitisse que mapeassem seus deslocamentos? A provocação contida nos primeiro e terceiro momentos proporciona manifestações de concordância inicial e posterior negação, por considerações bioéticas relacionadas à autonomia, não maleficência, justiça e beneficência na relação da saúde como prestadora de serviço.

Nas discussões fomentadas pelo assunto, é possível ver uma repetição de termos que se associam à assertividade para a pergunta. Apenas P7 se nega a registrar um dispositivo de monitoramento no primeiro momento. Novamente, os códigos mais apresentados são “bloqueio”, “objetivo”, “preservação”, “princípios éticos” e “benefícios”, em representação antagônica de respostas.

Embora pareçam códigos divergentes, há maior convergência com os demais relatos dos participantes com a assertiva de permissão do controle, desde que seja possível interrompê-lo de acordo com sua vontade. Durante o segundo momento, apontam-se questões sobre economicidade da tecnologia na saúde e da força social para implementação de inovações. Faz-se relevante mencionar as discussões sobre a compreensão pessoal dos direitos dos usuários de saúde no Brasil e também de suas implicações na telemedicina e demais ferramentas. Resguardar a relação profissional e preservar o direito de acesso dos usuários, além de elaborar legislação própria, é necessidade premente^(26)^.

Os participantes da pesquisa no terceiro momento partem do princípio que exista um arbítrio individual que sobreponha o bem-estar coletivo. Conforme considerado anteriormente, em países que possuem relações políticas mais fechadas, os cidadãos foram condicionados ao cumprimento das estratégias determinadas, seja por imposição de multas, desemprego ou prisão^(27)^.

Nas discussões finais, a análise do questionário pós-seminário mostra uma predominância de códigos assertivos, entre eles os termos “pactuação prévia”, “proteção de dados”, “bem coletivo” e “direitos”. A palavra “individualidade” e “direito” aparece citada nas negativas dos participantes, sendo que esses são os mesmos que declararam não aceitar a instalação de aplicativos de rastreamento no questionário do primeiro momento.

Estudos recentes da análise da legitimidade jurídica do uso das referidas tecnologias à luz da bioética marcam a necessidade de delimitação do tempo de uso dos aplicativos e eliminação de dados. De acordo com Ram e Gray^(28)^, é justamente a falta de tempo definido para encerramento do monitoramento do dito epidemiológico, que permite apropriação de dados por terceiros, invasões *hackers* e usos diversos para fins que não os previamente autorizados.

Morley *et al*.^(29)^ consideram que o problema precisa responder a questões que foram elencadas pelos participantes do estudo: a duração do procedimento; a efetividade do mesmo; a voluntariedade da participação; a finalidade da coleta dos dados; e o tempo de disponibilidade destes dados. Leva-se em consideração a perspectiva de que os dados possam ser deletados de acordo com a vontade do indivíduo, que seria o chamado “descomissionamento”.

Os participantes da pesquisa evocam a atuação profissional não para embasar o novo método tecnológico proposto em caráter de urgência, mas como se considerassem os métodos anteriores mais suaves e menos invasivos do que o rastreamento por aparelhos.

No terceiro momento, a sustentação de uma posição de desconfiança calcada na preservação de liberdades e direitos dos pacientes contrapõe a resposta inicial da maioria: não instalar o aplicativo de monitoramento e não recomendá-lo, dito por P2, P3, P4, P5, e P7. A abordagem remete ao uso prático da ética utilitarista e principialista. Na primeira, pela proteção de princípios que não estão relacionados a uma lógica preestabelecida, mas sim moldada caso a caso com base em moral individual e vivências pessoais. É o caso de quando os códigos mostram que o profissional se preocupa com a exposição pensando primeiro em si, na sua ação cidadã, para em seguida fazer uma interface profissional.

A ética utilitarista aparece na maximização do bem-estar a partir do sentimento de justiça universal. Quando as menções no seminário passam a sensação de iniciativa imposta, de autoridade governamental, os participantes optam pela repressão em construções que perpassam valores profissionais da bioética, como direitos do paciente e direitos do ser humano^(30-32)^.

As categorias apresentadas demonstram que os estudantes apresentam uma propensão à colaboração coletiva face à pandemia. No decorrer do segundo momento e com a culminância do terceiro, questões de ordem profissional, como segurança de dados de prontuários e outros sistemas já utilizados, concebidos como “não seguros”, fazem com que esses apresentem ressalvas ao uso dos dispositivos em perspectiva também profissional.

### Limitações do estudo

Embora o estudo tenha como objeto de trabalho um grupo focal como forma de captar as percepções em uma área que está em franca discussão, é necessária a ampliação da discussão para outros grupos, com atores sociais também diretamente impactados. Devem-se analisar as referidas percepções não só em grupos profissionais que integram programas de alta qualificação educacional, contemplando também a diversidade da nossa sociedade.

### Contribuições para a área da enfermagem, saúde ou política pública

O estudo demonstra que a aceleração de mudanças na prática do uso de dispositivos de rastreamento na saúde, provocada pela pandemia de covid-19, suscita profundas questões bioéticas de privacidade, direito individual e relações estado-indivíduo. O confrontamento dessas questões com os participantes fez com que se aprofundasse a discussão conforme a realidade vivenciada, que se baseou em preocupações com a dinâmica da missão profissional. Demonstra-se a necessidade de debate constante do uso de dispositivos de tecnologia de rastreamento em saúde, passando o período crítico da pandemia, de forma que sejam tomadas as melhores condutas e o uso da tecnologia esteja sempre a favor da segurança e do bem-estar dos pacientes, usuários, profissionais, gestores do serviço e toda a sociedade.

## CONSIDERAÇÕES FINAIS

As tecnologias de rastreamento de indivíduos foram consideradas uma alternativa para prevenção da covid-19, e tão logo o mundo se deparou com transmissão fora de controle e a inexistência de meios mais efetivos de contato. A aplicação de tecnologia que monitora o indivíduo ou comete algum cerceamento de direitos individuais é vista com desconfiança, mesmo quando considerados os supostos benefícios para seu uso em massa. Quando confrontados com esse novo paradigma no rol de ações específicas da pandemia e uma posterior extensão da prática para outras atividades na saúde coletiva e na epidemiologia, há uma certa insegurança motivada por valores ligados à bioética tanto no âmbito pessoal como no profissional.

A partir da análise do material produzido pelo seminário, evidenciou-se a existência de preocupação com as consequências do uso de tecnologias de rastreamento de indivíduos por parte dos participantes após a apresentação de argumentação sobre o tema, ensejando protagonismo e autonomia pessoal, bem como controle do estado na prática para o trabalho em saúde. Dessa feita, os participantes saem da opinião pessoal sobre o tema para reflexão sobre as implicações na atuação profissional na saúde.

A covid-19 não foi a primeira pandemia e provavelmente não será a última vivenciada. O uso desse tipo de dispositivo nesse contexto trouxe aprendizagens que poderão ser utilizadas em outras situações de gravidade igual ou superior, sem grandes empecilhos. Seu uso deve ser estimulado, desde que a confidencialidade, o sigilo e a liberdade do direito de escolha sejam também assegurados e respeitados. Essa realidade demonstra a necessidade de desenvolvimento de novos mecanismos de preservação desses preceitos no uso de dispositivos de rastreamento.

## Data Availability

https://doi.org/10.48331/scielodata.CGLEQU
